# Selfish risk-seeking can provide an evolutionary advantage in a conditional public goods game

**DOI:** 10.1371/journal.pone.0261340

**Published:** 2022-01-21

**Authors:** Martina Testori, Hedwig Eisenbarth, Rebecca B. Hoyle

**Affiliations:** 1 School of Mathematical Sciences, University of Southampton, Southampton, The United Kingdom; 2 School of Psychology, Victoria University of Wellington, Wellington, New Zealand; Teesside University, UNITED KINGDOM

## Abstract

While cooperation and risk aversion are considered to be evolutionarily advantageous in many circumstances, and selfish or risky behaviour can bring negative consequences for individuals and the community at large, selfish and risk-seeking behaviour is still often observed in human societies. In this paper we consider whether there are environmental and social conditions that favour selfish risk-seeking individuals within a community and whether tolerating such individuals may provide benefits to the community itself in some circumstances. We built an agent-based model including two types of agent—selfish risk-seeking and generous risk-averse—that harvest resources from the environment and share them (or not) with their community. We found that selfish risk-seekers can outperform generous risk-averse agents in conditions where their survival is moderately challenged, supporting the theory that selfish and risk-seeking traits combined are not dysfunctional but rather can be evolutionarily advantageous for agents. The benefit for communities is less clear, but when generous agents are unconditionally cooperative communities with a greater proportion of selfish risk-seeking agents grow to a larger population size suggesting some advantage to the community overall.

## Introduction

### Selfishness and risk preferences

Biological selfishness has been long discussed as an adaptive trait in both humans and animals [1]. Selfishness, defined as the gain of one’s fitness at the cost of others’, provides an advantage in the ‘survival of the fittest’, where genes that are passed on to the next generation in greater numbers become more frequent in the population. Selfish genomes have also been found to be a crucial element for evolutionary change and innovation in individuals [2]. Selfishness in this biological sense is intrinsically connected to fitness and evolutionary advantage. However, selfishness in the social sense may be selected against: both empirical and theoretical work have demonstrated that cooperative behaviour can evolve and be sustained in communities through mechanisms such as reciprocity, network structures, reputation systems, gossip and both peer and institutional punishment, or through kin or group selection [3–9].

Risk-aversion has been proposed as an evolutionarily advantageous trait [10], and has been particularly investigated in contexts of variability of resource acquisition [11–13]. When faced with uncertainty and risky decisions, evolutionary psychologists suggest that aversion is the prudent and evolutionary stable strategy to adopt, even though risk-seeking behaviours might lead to higher payoff [14]. A recent study investigating the origin of risk-aversion suggested that ‘rare, high-risk, high-payoff events such as mating and mate competition could have driven the evolution of risk averse behavior in humans living in small groups’ [13](p.1). Moreover, Kolodny & Stern (2017) indicate that life-history, and ecological context can have significant impact on the selection of risk preferences [15].

The survival of the individuals depends not only on their individual traits, but also on their community context. The relationship between individual and group fitness can be controversial [16]. In the specific case of selfishness, self-enhancing traits are usually detrimental for the growth of the community as a whole. On the other hand, the evidence for the impact of risk preferences on group fitness is more mixed [17, 18].

In this paper we are interested in selfish and risk-seeking behaviour where there might plausibly be a conflict between individual and group interests.

### Psychopathic traits as evolutionarily advantageous

Cleckley [19] described the psychopathy construct as characterised by a constellation of personality traits, including lack of remorse, guilt and fear, poor impulse control, sensation-seeking, emotional detachment and impairment in building stable relationships, as well as high levels of manipulativeness, selfishness, low empathy and callousness, that can be expressed on a continuous scale [20]. Selfishness and risk-seeking behaviour are two core characteristics of psychopathy, which has been described as evolutionarily adaptive [21]. This postulated evolutionary advantage could be driven by the ability of individuals high on psychopathy to be ‘cheater-hawks’, i.e. individuals who use manipulation and deception to exploit cooperation, but also adopt intimidation and aggression to achieve their goals. Previous studies report contradictory results on whether psychopathic traits are evolutionarily adaptive, or whether they are harmful variations of human personality [22, 23]. Several scholars have argued the case for an evolutionary advantage of psychopathy [23–29]: De Silva et al. discussed the adaptive role of some of the main features of psychopathy (such as thrill-seeking behaviours, low fear and unresponsive stress response) in hostile psychosocial environments [23]; and recent findings also support the conceptualisation of psychopathy as an adaptive strategy to cope with harsh experiences [30]. Based on key traits of the construct (e.g., manipulativeness, exploitativeness, deceptiveness, lack of fear and empathy, and superficial charm), Krupp et al. also argued that psychopathy is not a disorder but an ‘evolved life-history strategy’ [31].

Medjedović et al. (2017) examined the relationship between psychopathy and fitness, represented as the ability to reproduce offspring, considering the moderating role of the environment [32]. Their data showed that some aspects of psychopathy (interpersonal and affective sphere) correlate positively with individuals’ fitness, leading to more offspring in the future, while other aspects such as impulsivity and recklessness were negatively correlated with reproductive success, suggesting a disadvantage for future survival and procreation. On a cross-sectional level, there is evidence that some aspects of psychopathy, namely the fearless-dominant ones, are related to higher professional success [33–35].

In this paper, we will focus on the fitness costs and benefits of selfishness and risk-taking by individuals embedded in a community experiencing different environmental conditions, inspired by the example of psychopathy. The psychopathy construct is complex and characterised by a multitude of traits. In this model, we focus on only two of these traits. More extensive models including a variety of those traits should be built to study psychopathy in its entirety as well as to differentiate the contribution of its variants [36].

### Our model

We build an agent-based model (ABM) to investigate whether selfish and risk-seeking traits can be beneficial not only to the individual but also to the community.

We consider the evolution of a population where two types of agents (henceforth phenotypes)—**selfish risk-seekers (S)** and **generous risk-averse (G)**—evolve in different environmental conditions. Our model comprises two main stages: we consider a population in which agents (1) gather resources from the environment for their own well-being, and (2) decide how much to contribute to the community’s wealth by donating part of their gathered resources. Environmental scenarios are represented here as both natural conditions (such as the availability of resources per agent) and social conditions (such as the cost that either the agent or the community has to pay for anti-social behaviours). By implementing this framework over a number of generations, we aim to disentangle the beneficial (or harmful) contribution of selfish risk-seekers in a community over an extended period of time.

Building upon previous research, we hypothesise that when environmental conditions become scarcer for the survival of the community, the adaptive side of selfish risk-seeking behaviours will prevail, leading to a community mostly composed of agents with selfish and risk-seeking attitudes.

## Materials and methods

**Diagram 1:**
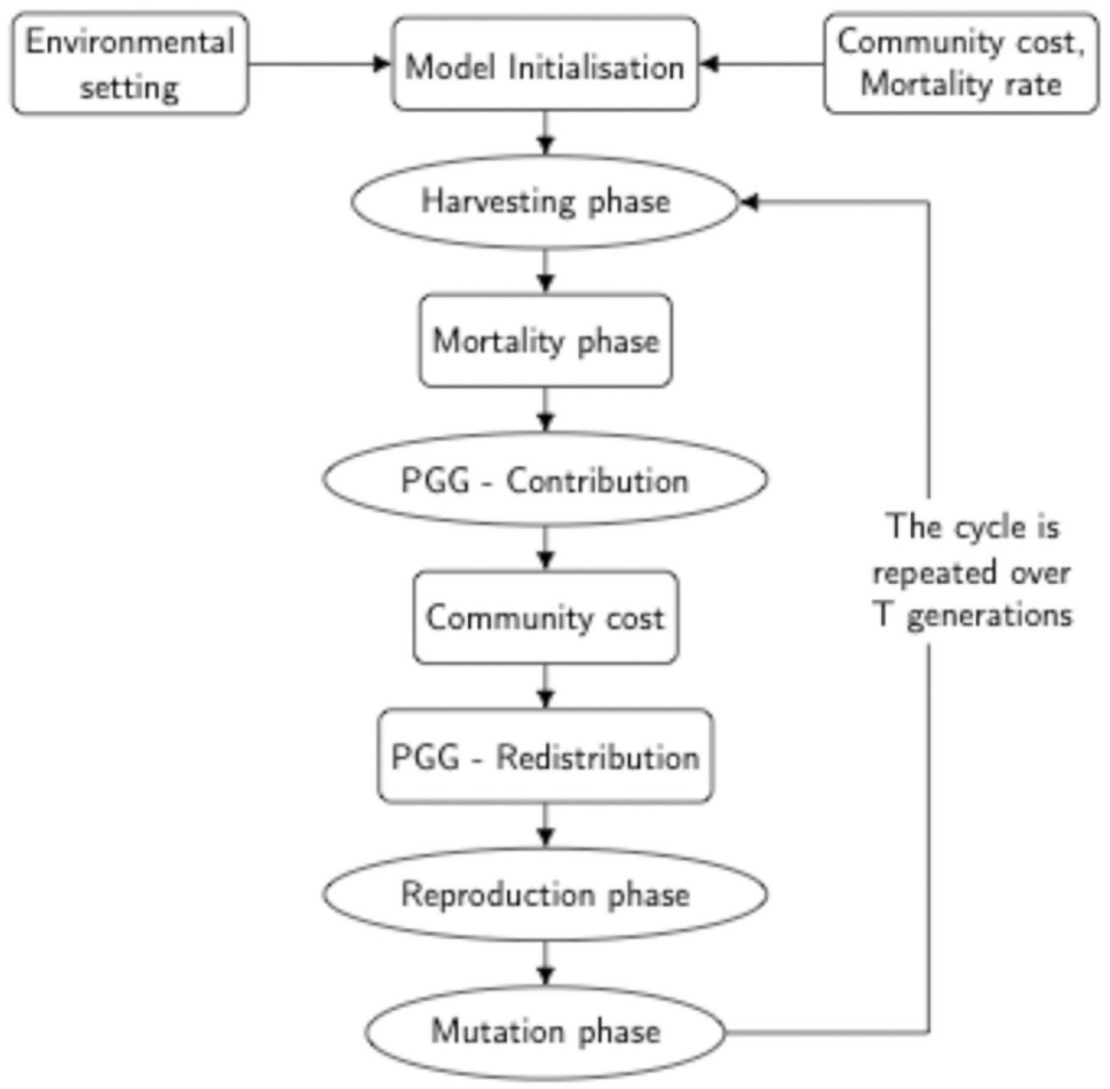
Flowchart of the model dynamics.

The model consists of four main stages: (1) harvesting, (2) public contribution (Public Goods Game, [37]), (3) reproduction and (4) mutation, as illustrated in Diagram 1.

The initial population is composed of *N*_0_ agents, whereof *S*_0_ = 10% are selfish risk-seekers. Full details of the model can be found below and in the section S1 in S1 File.

### Harvesting

First, agents gather resources from the environment. The environment provides a fixed amount of resources per generation *e*_*tot*_, independent of the population size. Each agent can harvest an equal proportion of the total amount *e*_*t*_, which varies according to the number of agents in the population. Each agent cannot harvest more than a certain amount per generation *e*_*max*_. During this first step, agents perish with a certain probability *m* as a consequence of engaging in the risky action of harvesting (mortality phase). Since risk-averse agents do not engage in risky actions, they are not affected by the mortality phase. Risk-seekers always harvest *h*_*S*_ = 80% of the available resources, while the risk-averse gather *h*_*G*_ = 50% of the resources offered by the environment.

### Public goods game

Second, agents participate in a Public Goods Game (PGG, [37]) in which they contribute a portion of their resources (*c*_*G*_, *c*_*S*_) to the communal pot. The total amount in the pot is multiplied by a constant factor *ρ* and redistributed equally among all agents, regardless of their initial contribution. Generous risk-averse agents contribute *c*_*G*_ = 100% of their resources (they are also later modelled as conditional cooperators), while selfish risk-seekers contribute only *c*_*S*_ = 20%. Before the redistribution of the resources, the community pays a cost (λ) proportional to the number of selfish risk-seeking agents (community cost phase). This represents the cost that selfish and risk-seeking agents impose on the community, through anti-social behaviours resulting from these personality traits, for example by breaking the rules or disrupting the community. The communal pot is then redistributed equally among all agents.

### Reproduction

Third, each agent reproduces proportionally to their fitness. The parent agent *i* dies at the end of the current generation and is replaced by a number *n*_*i*,*t*_ of offspring in the next generation as described in Eq 1.

$$\begin{array}{c} n_{i,t}=\{\begin{array}{cc} 0, & \text{if}\;f_{i,t}\leq s\\ \lceil \frac{o}{M-s}\left(f_{i,t}-s\right)\rceil , & \text{if}\;s<\;f_{i,t}<M\\ o, & \text{if}\;f_{i,t}\geq M \end{array} \end{array}$$ (1)

where *f*_*i*,*t*_ is the agent’s fitness, *M* = *s* * *o* + *s* are the necessary resources for an agent to reproduce the maximum number *o* of offspring, and *s* is the survival threshold: if the parent did not gather enough resources to meet *s*, they perish without offspring. The offspring’s phenotype (*p*_*j*_) is determined by a reproduction mechanism (*φ*_*pi*,*pj*_) which takes into account the phenotype expressed by the parent (*p*_*i*_). Although offspring typically inherit the phenotype of their parent in evolutionary game theory, here we use a reproduction mechanism where parental influences modify a background frequency of personality types in order to mimic the influence of genetic (*ϕ*) and behavioural (*ω*, *γ*) contributions from parents on offspring personalities within a broader societal context:

$$\begin{array}{c} \varphi_{G,G}=1-\phi +\omega , \end{array}$$ (2)

$$\begin{array}{c} \varphi_{G,S}=\phi -\omega , \end{array}$$ (3)

$$\begin{array}{c} \varphi_{S,S}=\phi +\gamma , \end{array}$$ (4)

$$\begin{array}{c} \varphi_{S,G}=1-\phi -\gamma . \end{array}$$ (5)

$$\begin{array}{c} 0\leq \varphi_{G,G},\varphi_{G,S},\varphi_{S,G},\varphi_{S,S}\leq 1 \end{array}$$ (6)

where *ϕ* represents the agents baseline propensity to reproduce selfish risk-seeking offspring; *γ* (0 ≤ *γ* ≤ 1 − *ϕ*) embodies the effect of having a selfish risk-seeking parent in the development of personality traits; while *ω* (0 ≤ *ω* ≤ *ϕ*) embodies the effect of having a generous risk-averse parent in the development of personality traits. The form of the reproduction rates is chosen to reflect that previous literature shows both genetic and environmental cues to have a strong influence on the development of psychopathic traits [38, 39]. The reproduction mechanism is such that a selfish risk seeker will reproduce a selfish offspring with probability *ϕ* + *γ* and a generous risk-averse agent will reproduce a generous offspring with probability 1 − (*ϕ* − *ω*) (see more details in the S1 File). Within these rules the phenotype of each offspring of a given parent is determined independently of those of any siblings. Each offspring inherits its parent’s reproduction probabilities which are then subject to mutation in the next step.

### Mutation

Lastly, we introduce a mutation in the reproduction probabilities. At each generation *t*, the reproduction probabilities of each agent are subject to a small mutation (*η*_*ϕ*,*t*_, *η*_*γ*,*t*_, and *η*_*ω*,*t*_) drawn from normal distributions with mean 0 and variances $\sigma_{\phi }^{2}$, $\sigma_{\gamma }^{2}$ and $\sigma_{\omega }^{2}$ respectively. Thus, the reproduction probabilities (now denoted *ϕ*_*t*_, *γ*_*t*_ and *ω*_*t*_ at generation *t*) evolve over generations as follows:

$$\begin{array}{c} \varphi_{G,G,t}=1-\phi_{t}+\omega_{t}=1-\phi_{t-1}-\eta_{\phi ,t}+\omega_{t-1}+\eta_{\omega ,t}, \end{array}$$ (7)

$$\begin{array}{c} \varphi_{G,S,t}=\phi_{t}-\omega_{t}=\phi_{t-1}+\eta_{\phi ,t}-\omega_{t-1}-\eta_{\omega ,t}, \end{array}$$ (8)

$$\begin{array}{c} \varphi_{S,S,t}=\phi_{t}+\gamma_{t}=\phi_{t-1}+\eta_{\phi ,t}+\gamma_{t-1}+\eta_{\gamma ,t}, \end{array}$$ (9)

$$\begin{array}{c} \varphi_{S,G,t}=1-\phi_{t}-\gamma_{t}=1-\phi_{t-1}-\eta_{\phi ,t}-\gamma_{t-1}-\eta_{\gamma ,t}, \end{array}$$ (10)

See S1 File for further details. The entire cycle is then iterated over *T* generations and, in order to obtain robust results, the model is repeated over 100 independent realisations. Simulations were carried out in MATLAB, version R2019a [40].

We carried out simulations for two different environments as measured by their ‘environmental offer’ (see Table 1): an abundant environment that provides enough resources for communities to flourish in most circumstances and a scarce environment in which survival is more challenging. The environmental offer values for these two environments were selected after investigating their effect on a population in which each agent reproduced offspring of the same phenotype without mutation (see section S2 in S1 File). While the abundant environment allowed the population to grow over their initial population size in most of the settings investigated, the scarce environment led the community to shrink, and sometimes even to extinction (see S1 File for more details). We also set the maximum amount of resources each agent could harvest per generation to be the environmental offer of the abundant environment.

**Table 1:** Overview of the model parameters and their values.

Level	Parameters		Range
Model			
	Initial population size	*N*_0_	250
	Number of generations	T	10^4^
	Model realisations	I	100
Environment			
	Environmental offer	*e*	Abundant: *e* = 4.5, Scarce: *e* = 1.2
	Total resources offered at each time step	*e*_*tot*_ = *e* * *N*_0_	1.2,4.5*N_0_
	Agent resources available at generation t	*e*_*t*_ = *e*_*tot*_/*N*_*t*_	[0,10^4^] units
	Maximum amount of resources per agent	*c*_*max*_	4.5 units
Community			
	Community cost for S agents	λ	λ ∈ {0%, 50%, 100%}
	Public Goods Game (PGG) Multiplier	*ρ*	*ρ* = 1.5
	Initial proportion of selfish agents in the population	*S*_0_	*S*_0_ = 10%
Agent ≡ Phenotype *p* ∈ {*S*, *G*}			
	Harvesting rate	*h*_*p*_	*h*_*S*_ = 80%, *h*_*G*_ = 50%
	Contribution rate (decimal format)	*c*_*p*_	*c*_*S*_ = 0.2,*c*_*G*_ = 1
	Mortality rate	*m*_*p*_	*m*_*G*_ = 0%; *m*_*S*_ ∈ {0%, 25%, 50%}
	Reproduction probability	*φ*_*p*,*p*_	[0, 1]
	Maximum number of offspring	*o*	*o* > 0, *o* = 10
	Survival level	*s*	*s* ≥ 0, *s* = 1
	Initial probability of each agent to reproduce a selfish offspring	*ϕ*	0.1
	Variance of the mutation distribution for *ϕ*	$\sigma_{\phi }^{2}$	0.01
	Initial component embodying the effect of having a generous parent	*γ*	0.01
	Initial component embodying the effect of having a selfish parent	*ω*	0.01
	Variance of the mutation distribution for both *γ*, *ω*	$\sigma_{\gamma }^{2},\sigma_{\omega }^{2}$	0.01

The main parameters used in the model and their values are presented in Table 1.

## Results

Three models are presented in the following sections. After the baseline model described above, we implement two other features (conditional cooperation for generous agents and individual cost for risk-seekers) to better represent dynamics in the real world. One component is implemented at a time to investigate the effects of each feature separately, and have a clear understanding of how each one affects the population’s evolution. In the SI, we also report the dynamics for (1) a simple model with perfect transmission, in which agents always reproduce offspring of their own phenotype (see section S3 in S1 File); and (2) the baseline model with no mutation, meaning that generous agents always have a 91% chance of reproducing generous risk-averse offspring, while selfish agents always have an 11% chance of reproducing selfish risk-seeking offspring (see section S4 in S1 File).

The results reported below describe the equilibrium state of the population after 10^4^ generations. For an overview of the evolution over time, see the S1 File.

### Baseline model

The results reported for this model refer to an abundant environment (*e* = 4.5), since no qualitative differences were observed between the two levels of resources: although the population size varied between the two environments, the percentage of selfish and generous agents differed by no more than 1.5% (see section S5.1 in S1 File for an overview of the outcomes in both environments).

**Fig 1:**
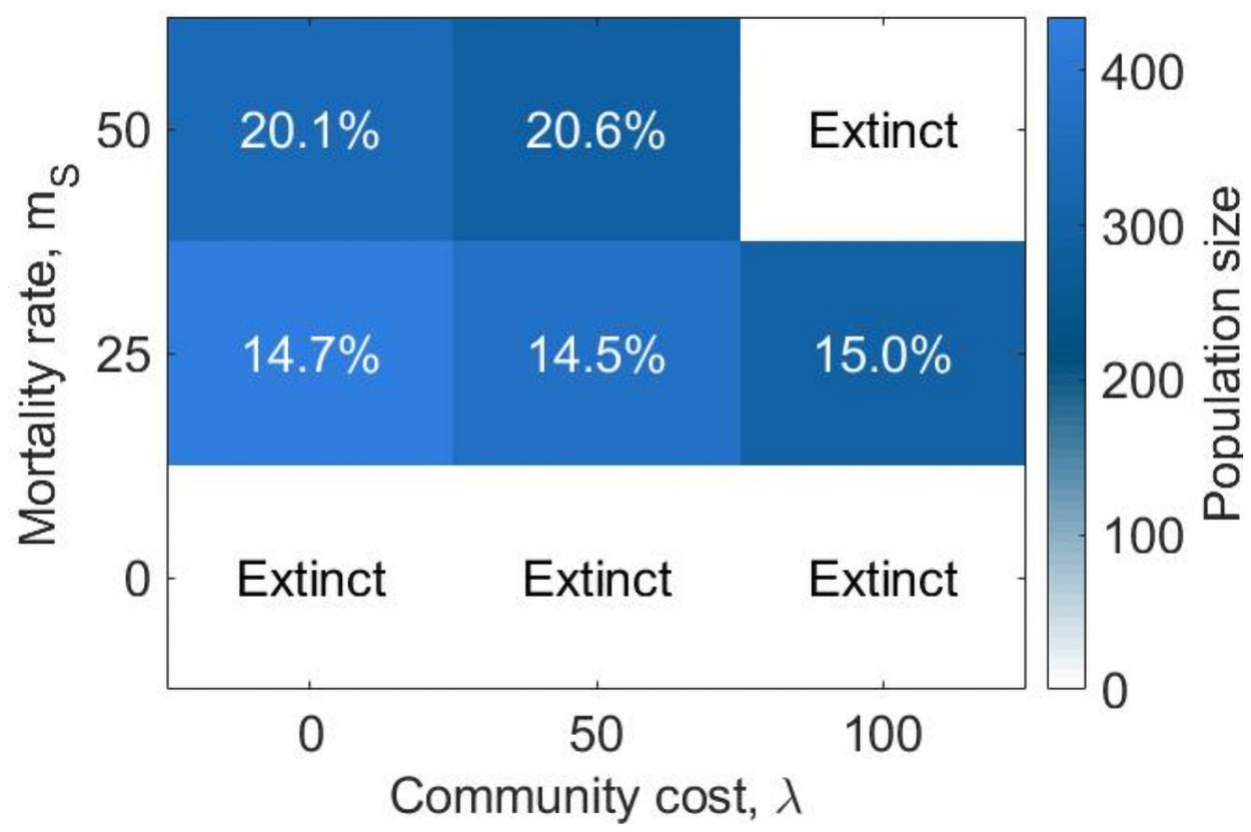
Equilibrium state for populations in an abundant environment (*e* = 4.5) when varying the mortality rate for selfish risk-seeking agents and the community cost. The color shade represents the population size at equilibrium, while the percentages represent the proportion of generous risk-averse agents in the population at equilibrium. White tiles represent the conditions in which the population reached extinction by the 104 generation. While most populations survive and are mostly composed of selfish risk-seekers when the mortality rate is greater than zero, populations reach extinction if no mortality rate is imposed on selfish risk-seeking agents.

Fig 1 shows the equilibrium population size and composition for various values of the mortality rate for selfish risk-seekers (*m*_*S*_ ∈ {0%, 25%, 50%}) and the community cost (λ ∈ {0, 50, 100}). The parameter that most strongly affects the evolution is the mortality rate for selfish risk-seekers during harvesting: when no mortality rate is introduced, the population initially grows very fast owing to the success of selfish risk-seekers and rapidly becomes too large for the environment to sustain leading to extinction (S10 and S11 Figs in S1 File). By introducing a small mortality risk for risk-seekers (*m*_*S*_ = 25%), the population survives, reaching a stable equilibrium. Selfish risk-seeking agents comprise the majority of the community (roughly 85%), leaving little space for generous risk-averse agents. The effect of the community cost on the dynamics also becomes visible: the larger the cost the community has to pay to sustain selfish agents, the fewer agents can be sustained at equilibrium. This is even more evident when the mortality rate is increased to 50%. In this case, the population survives and reaches equilibrium only for zero or medium (λ = 0, 50) community cost. When the community cost is increased to λ = 100, the population perishes before the 10^4^ generation.

Finally, results show that when the percentage of selfish risk-seeking agents is greater communities reach a larger size (Fig 1). This suggests that selfish risk-seeking behaviour supports larger communities and so can be beneficial not only to the selfish risk-seekers themselves, but also to the wider community.

**Fig 2:**
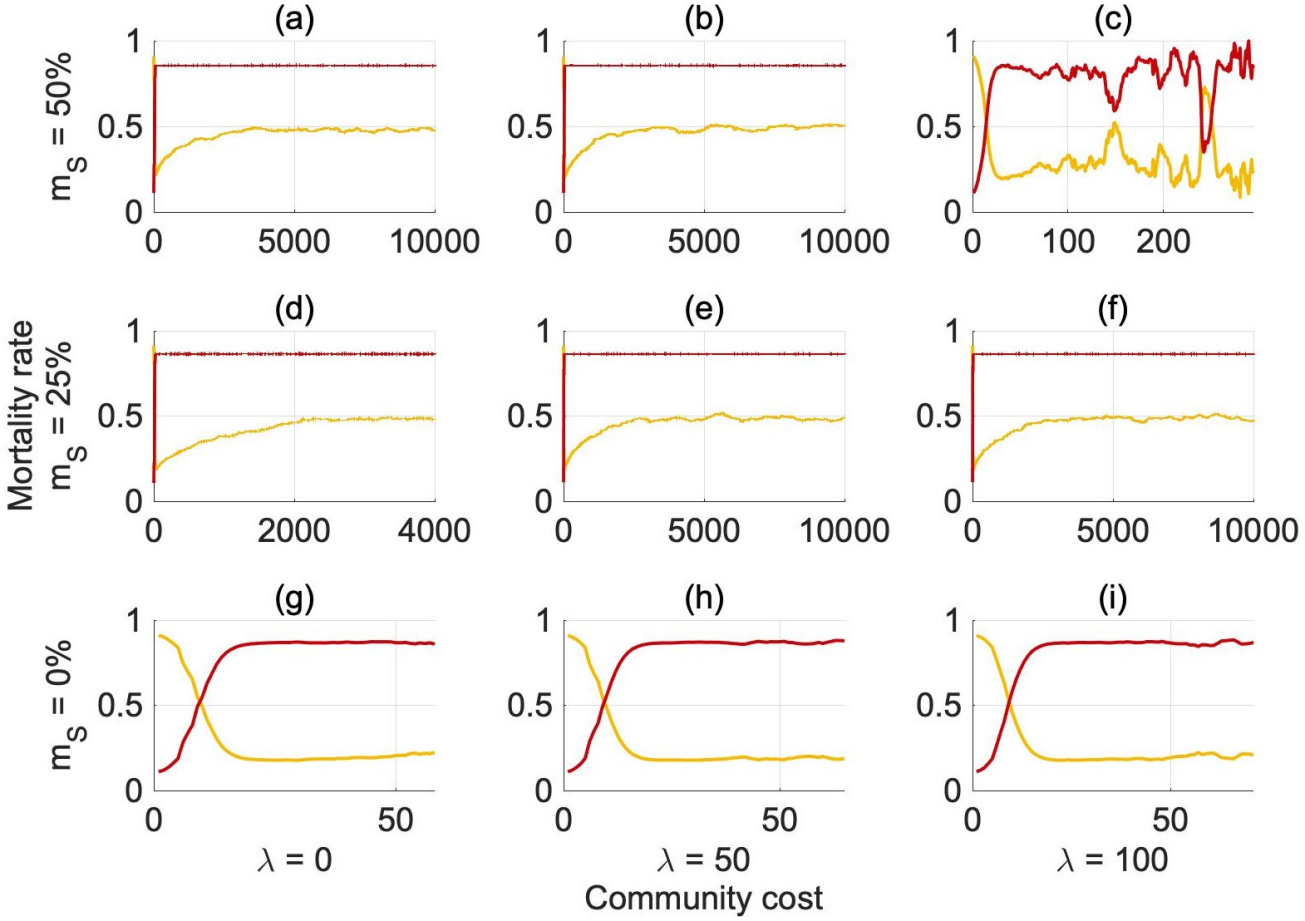
Average evolution of the combined reproduction rates *φ_S,S_*, *φ_G,G_* over generations in an abundant environment (*e* = 4.5). Redline: *φ_S,S_*, Yellowline: *φ_G,G_*. *φ_G,G_* = 1(0)/*φ_S,S_* = 1(0) means that a generous risk-averse/selfish risk-seeking agent has a 100(0)% chance of reproducing generous risk-averse/selfish risk-seeking offspring.

**Fig 3:**
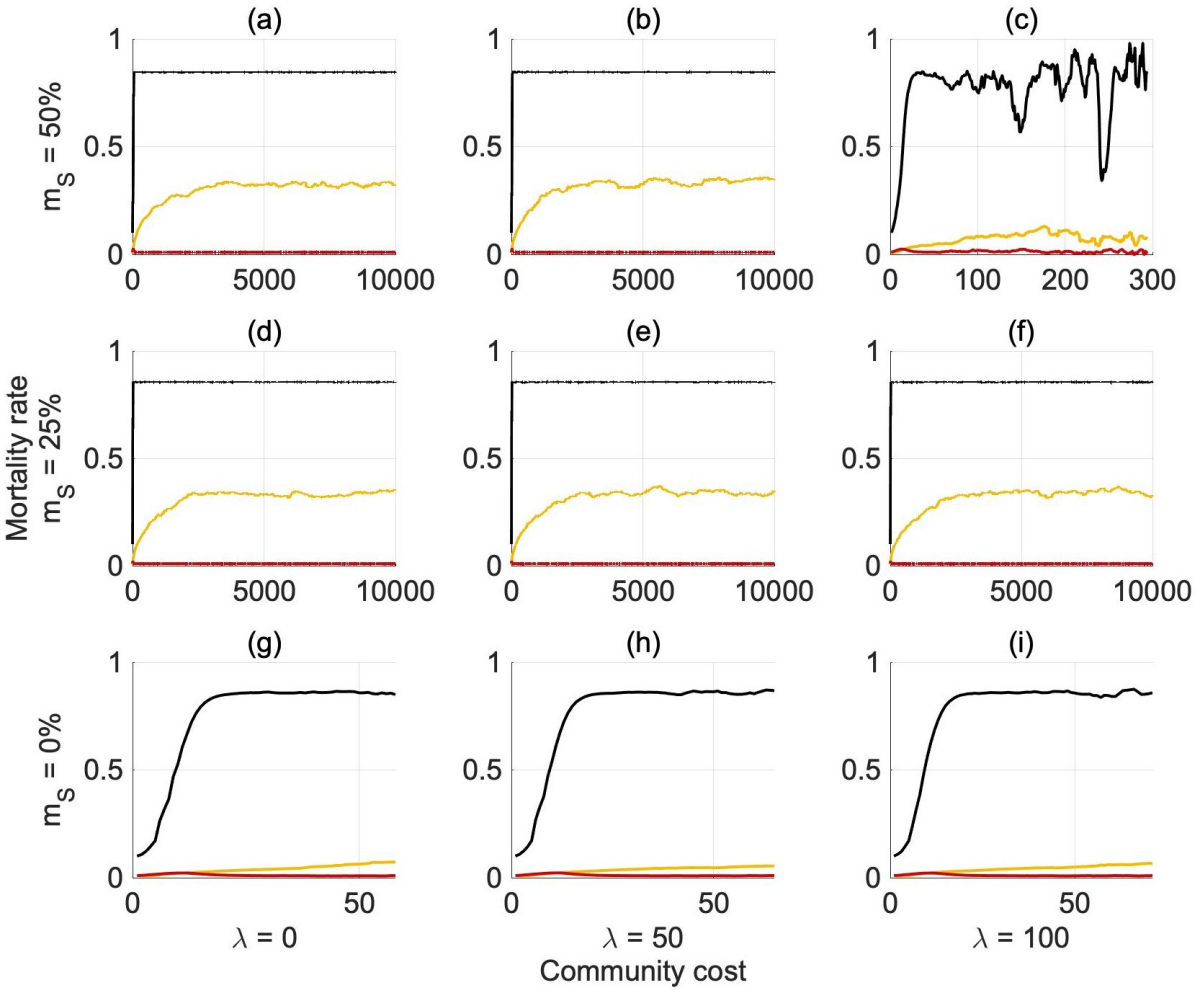
Average evolution of the separate parameters (*ω*, *γ* and *ϕ*) regulating the reproduction mechanism over generations in an abundant environment (*e* = 4.5). Redline: *γ*, contribution of selfish risk-seeking parent to the probability of reproducing selfish risk-seeking offspring, Yellowline: *ω*, contribution of generous risk-averse parent to the probability of reproducing generous risk-averse offspring, **Black** line: *ϕ*, population baseline contribution to the probability that agents will reproduce selfish risk-seeking offspring (independent of parental phenotype).

Fig 2 shows the evolution of the reproduction rates *φ*_*G*,*G*_ and *φ*_*S*,*S*_ over generations, while the separate evolution of *ϕ*, *γ* and *ω* is presented in Fig 3 in an abundant environment (see S12 and S13 Figs in S1 File for the scarce environment). The baseline likelihood of reproducing selfish offspring *ϕ* reaches a very similar equilibrium value independent of the scenario considered (Fig 3).

For *m*_*S*_ = 0%, only the first generations are shown as the population dies out after about 70 generations. Nonetheless, the parameter *ϕ* (and the reproduction rates *φ*_*G*,*G*_, *φ*_*S*,*S*_) has already reached the stable equilibrium, *ϕ* ∼ 90% (*φ*_*G*,*G*_ ∼ 0.25; *φ*_*S*,*S*_ ∼ 0.85) that is consistent with the other scenarios (see Figs 2(g)–2(i) and 3(g)–3(i)). When the mortality risk is set at *m*_*S*_ = 50% and the community cost is equal to 100%, the equilibrium seems to be approached but there is strong variability (Figs 2(c) and 3(c)). This is due to the noise in the evolution of the population in the most critical condition, which leads the population to go extinct in several of the independent realisations.

Since *γ* remains constant around zero in all scenarios (Fig 3), the probability of selfish agents reproducing selfish offspring is controlled by the component *ϕ*. Thus, selfish risk-seeking parents do not have a higher probability of reproducing selfish risk-seeking offspring compared to generous risk-averse agents, indicating that the main factors influencing the reproduction of selfish risk-seekers are general to the whole population (*ϕ*: population baseline contribution to the probability that agents will reproduce selfish risk-seeking offspring) rather than specific to the influences of the selfish parental phenotype (*γ*: contribution of selfish risk-seeking parents to the probability of reproducing selfish risk-seeking offspring). Conversely, the reproduction probability for generous agents evolves over time according to the exogenous conditions (i.e., mortality rate and community cost) implemented. As is visible in Fig 3, *ω*, the influence of generous parental phenotype on reproduction rates, evolves over generations, reaching a stable equilibrium around generation 400 (unless the population reaches extinction first). When the mortality rate is set to either 25 or 50%, generous agents become more likely to reproduce generous offspring over time. Thus, the mean equilibrium probability for generous agents of passing on their own phenotype varies from 20% to 50% according to the parameter settings. The evolution of these three parameters is driven by the dominant phenotype in the population. If selfish risk-seekers reproduce more offspring, then the reproduction rate evolves in such a way as to favor selfish risk-seekers in future generations (and vice-versa for generous risk-averse agents). *ϕ* clearly shows this evolution over time (see for example Fig 3 where selfish risk-seekers clearly dominate the population).

Overall, the results suggest that selfish agents are mostly favoured. The large advantage of selfish risk-seeking agents is driven by their ability to gather more resources and their tendency to share a smaller percentage of them with the community, compared to generous agents. Thus the community cost is almost entirely supported by generous agents who contribute 100% of their resources to the public pot. Therefore, even though risk-seekers might perish during the first phase of the model, if they survive they are assured of success in the later stages. This evolution is not realistic as agents engaging in anti-social and criminal activities are usually either isolated from the community or punished for their actions, for example by imprisonment. A cause of the strong advantage of selfish risk-seekers is rooted in the strategy of generous agents, who are modelled as pure cooperators who do not adapt to the environment or to the other agents in the community. This behaviour is relatively uncommon in experiments or in everyday life. To address these issues, the next section presents a model in which generous agents adapt their contributions to the community, playing a conditionally cooperative strategy; and the following section includes an individual cost for selfish risk-seekers.

### Conditional cooperation

In this section, generous risk-averse agents are modelled as conditional cooperators: they start by adopting a cooperative strategy in the first generation, *c*_*G*,0_ = 1, and they then change their strategy according to how other agents acted in the previous generation. Thus generous risk-averse agents adapt to their social environment, adjusting their strategy to others’ behaviour:

$$\begin{array}{c} c_{G,t}=\{\begin{array}{cc} 1, & \text{if}\;t=0\\ \frac{1}{N}\sum_{j=1}^{N}c_{j,t-1}, & \text{if}\;t\geq 1. \end{array} \end{array}$$ (11)

**Fig 4:**
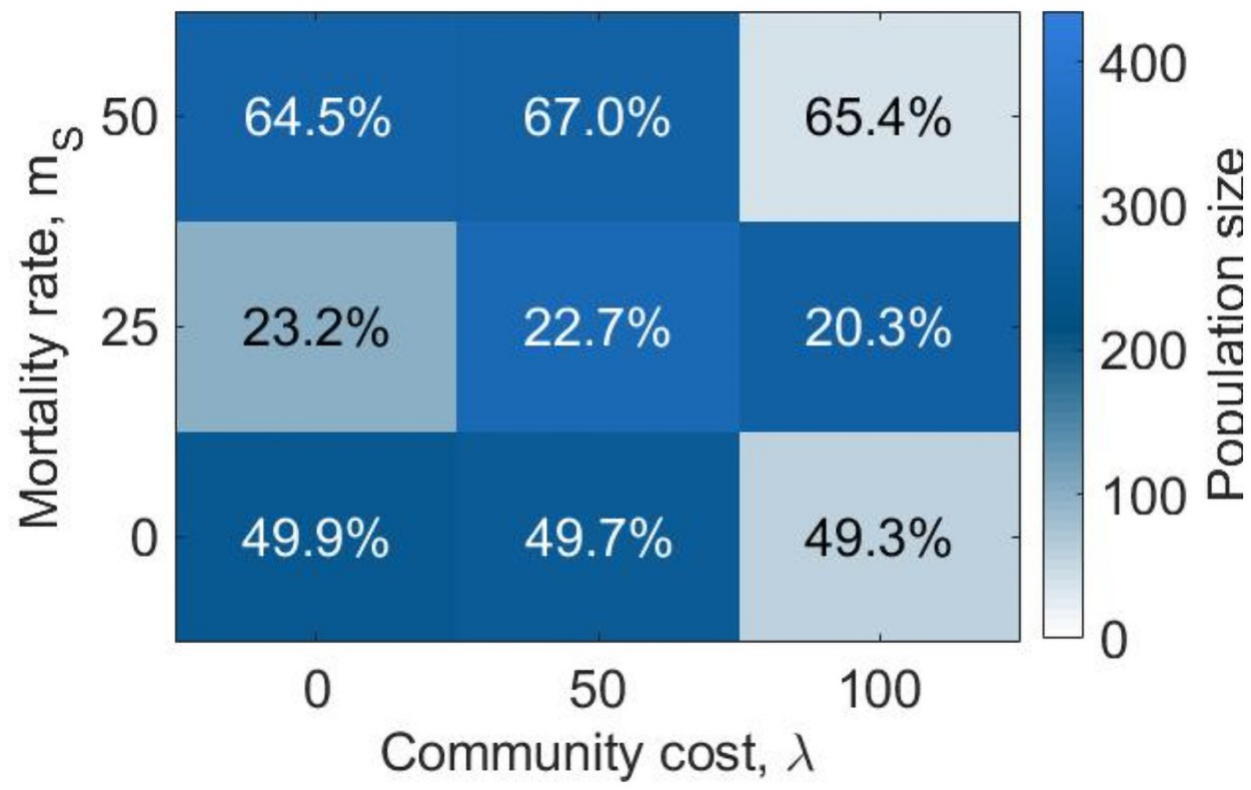
Equilibrium state for populations in an abundant environment (*e* = 4.5), when generous agents behave as conditional cooperators. The color shade represents the population size at equilibrium, while the percentages represent the proportion of generous risk-averse agents in the population at equilibrium. Populations do not reach extinction regardless of mortality rate or community cost. At 0% or to 50% mortality rate the majority of the population comprises generous risk-averse agents, while the opposite happens at 25% mortality rate.

**Fig 5:**
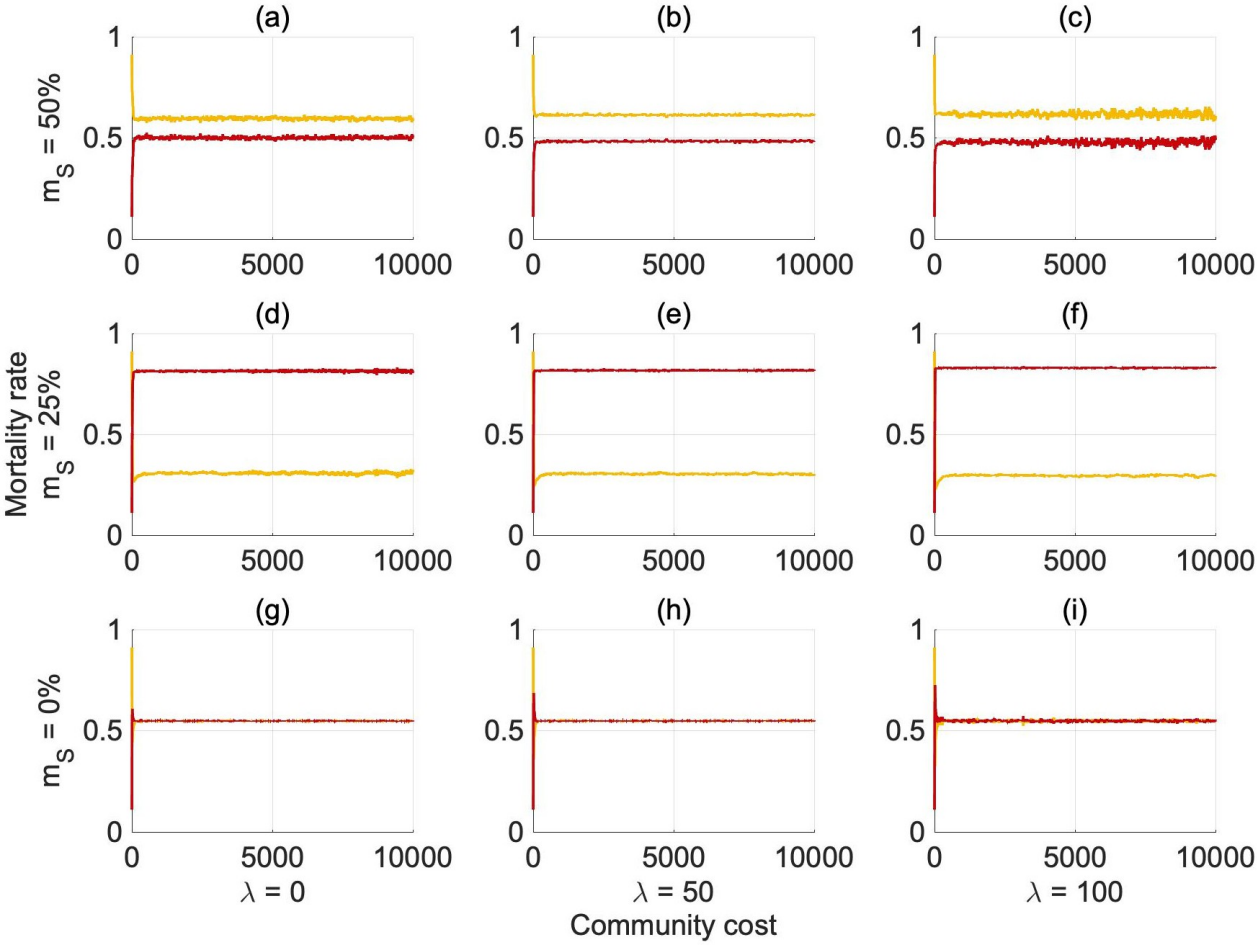
Average evolution of the combined reproduction rates *φ_S,S_*, *φ_G,G_* over generations when generous agents behave as conditional cooperators and the environment is abundant (*e* = 4.5). Redline: *φ_S,S_*, Yellowline: *φ_G,G_*. *φ_G,G_* = 1(0)/*φ_S,S_* = 1(0) means that a generous risk-averse/selfish risk-seeking agent has a 100(0)% chance of reproducing a generous risk-averse/selfish risk-seeking offspring. At 0% mortality rate, both selfish and generous agents have a 50% chance of passing on their own phenotype to their offspring. At 50% mortality rate, generous agents have a high probability of passing on their own phenotype to their offspring (*φ_G,G_* ~ 65%), while less than half of selfish agents reproduce selfish offspring.

That is, the contribution of generous risk-averse agents is equal to the average contribution of the previous generation. Populations survive in all conditions when the environmental offer is abundant (see Fig 4 for the population and Fig 5 for the evolution of the reproduction rates). When resources are scarce, they survive in most of the scenarios (except for *m*_*S*_ = 25%, λ = 0 and *m*_*S*_ = 0%, 50%, λ = 100, see S16 Fig in S1 File). The evolution of the population is similar in the scarce and abundant environments and the percentage of selfish and generous agents at equilibrium are almost identical.

Results suggest that, while the benefit provided by selfish risk-seekers is clear in the baseline model, their contribution to the community becomes less clear when generous agents act as conditional cooperators. Enabling generous agents to adjust to the surrounding environment by responding to past generations allows the community to survive and to reach a population composition where generous agents are sometimes favoured over selfish risk-seekers (*m*_*S*_ = 50%). This can also be observed by looking at the reproduction rates of the two phenotypes: generous risk-averse agents are more likely to reproduce their own phenotype than selfish risk-seekers when mortality rate is high (Fig 5, *m*_*S*_ = 50%), suggesting that generous risk-averse agents are more beneficial to the community compared to selfish risk-seeking ones. Since the only parameter altered is the strategy of generous people in the PGG, it is evident that modifying generous agents’ contribution in response to the group average behaviour is the driving element of these changes in the evolution. Thus, communities can typically survive in both scarce and abundant environmental conditions (except in the scarce environment when the mortality and community cost are high), and in some settings generous risk-averse agents can evolve to comprise half (or slightly more than half) of the population, if they adopt a conditional cooperation strategy.

### Conditional cooperation and individual cost for selfish risk-seekers

As discussed in the first section of the results, after the harvesting phase selfish risk-seekers are successful agents, who gather more resources and share a smaller portion of what they gather with the community, compared to generous agents. However, in reality people who engage in anti-social and disruptive behaviours often incur additional costs, either social costs (being ostracised from the rest of the population) or institutional costs (for example being incarcerated) [41, 42]. To reflect this in the model, we include an individual cost that selfish risk-seekers have to pay after the resources have been redistributed in the PGG. The fitness of selfish risk-seekers at the end of the generation is adjusted as follows:

$$\begin{array}{c} f_{i,t}=\frac{C_{t}\rho }{N_{t}}+r_{i,t}\left(1-c_{p_{i}}\right)-\alpha , \end{array}$$ (12)

where *α* represents the individual cost risk-seekers have to pay and is set to be a fixed amount per agent. Different values of *α* were analysed, starting from a high value of 1, which is equal to the resources necessary to survive, to a low cost of 0.1. Results are shown here for *α* = 0.1 (see section S5.3 in S1 File for an overview of the impact of different costs, S24 and S25 Figs in S1 File). The inclusion of this cost presents a more realistic representation of dynamics, that does not automatically favour selfish risk-seeking attitudes over generous risk-averse ones.

**Fig 6:**
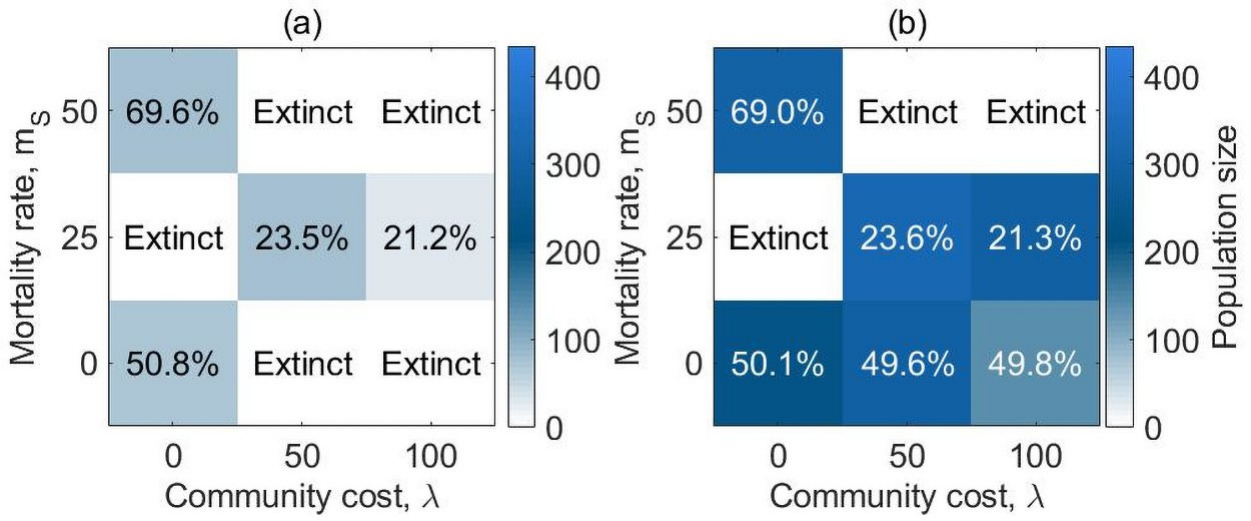
Equilibrium state for populations in (a) a scarce (*e* = 1.2) and (b) an abundant (*e* = 4.5) environment, when generous risk-averse agents act as conditional cooperators and selfish risk-seekers bear individual costs. The color shade represents the population size at equilibrium, while the percentages represent the proportion of generous risk-averse agents in the population at equilibrium. White tiles represent the conditions in which the population reached extinction by the 10^4^ generation. At 25% mortality rate, populations reach a stable equilibrium and the majority of the community is composed of selfish agents unless the community cost is set to 0. At 50% mortality rate, population reaches extinction before the 10^4^ generation when a community cost is present (λ = 50, 100) but survives in its absence (λ = 0) and 70% of the population expresses generous risk-averse attitudes.

Fig 6 shows the population size and composition at equilibrium (after 10^4^ generations), when resources are (a) scarce and (b) abundant. Comparing Fig 4 to Fig 6(b) shows that on introducing an individual cost for selfish risk-seeking agents communities fail to survive when the mortality rate is high (*m*_*S*_ = 50) and there are costs for the community (λ = 50, 100) or when there are no costs for the community and the mortality rate is set to 25. Thus when selfish risk-seeing agents bear an additional cost, the population is more likely to perish before the 10^4^ generation. The surviving communities have a similar proportion of selfish risk-seekers across the two models.

Furthermore, while in the previous models the environmental offer typically only affected the size of the equilibrium population, here we find an impact of the resources available on whether populations survive or not (Fig 6(a) and 6(b)). In the absence of mortality risk (*m*_*S*_ = 0%), communities survive when the environment is abundant, but they perish in most cases when the resources are scarce (*m*_*S*_ = 0%, λ = 50, 100) only surviving if no community costs are introduced.

**Fig 7:**
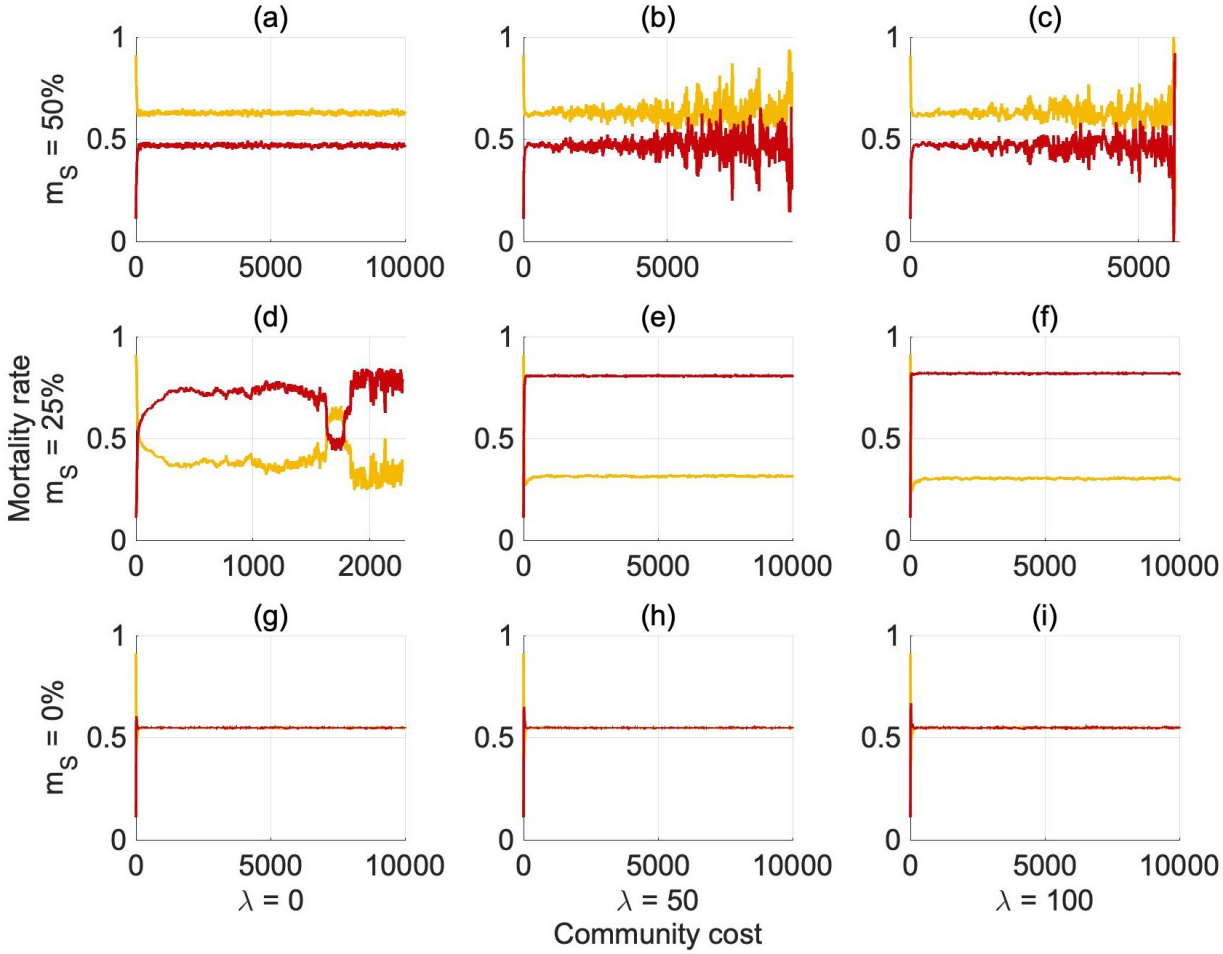
Average evolution of the combined reproduction rates *φ_S,S_*, *φ_G,G_* over generations, in an abundant environment, when generous risk-averse agents act as conditional cooperators and selfish risk-seekers bear individual costs. Redline: *φ_S,S_*, Yellowline: *φ_G,G_*. *φ_G,G_* = 1(0)/*φ_S,S_* = 1(0) means that a generous risk-averse/selfish risk-seeking agent has a 100(0)% chance of reproducing a generous risk-averse/selfish risk-seeking offspring. At 25% mortality rate, selfish risk-seeking agents are more likely to be reproduced, while generous risk-averse behaviours are favoured when the mortality is high *m_S_* = 50%.

The environmental resources do not impact the evolution of the reproduction probabilities, therefore Fig 7 only reports the findings for an abundant environment (see S28 Fig in S1 File for the evolution in a scarce environment and S29 and S30 Figs in S1 File for the evolution of the separate parameters of the reproduction probabilities in both environmental conditions). The reproduction probabilities reach a stable equilibrium (Fig 7), confirming the results found for the conditional cooperation model (Fig 5).

## Conclusion

Our model focused on the evolution of a community composed of two different behavioural types: those exhibiting generous risk-averse attitudes and those with selfish risk-seeking behaviours. We investigated the evolution of the populations when environmental as well as social conditions are varied to mimic a harsher or a more benevolent scenario.

It has been widely shown in previous literature that in models assuming fixed population size, defectors can survive without becoming extinct [43, 44]. In contrast, when the population size can fluctuate, defectors cannot always sustain themselves on their own, thus cooperators must be present, and selection must be sufficiently strong in order to avoid extinction [45]. It has also been shown that cooperators can prevail over defectors in a PGG if the population density depends on the average population payoff [46].

Our simulations suggest that selfish risk-seeking behaviours can be favoured over generous risk-averse attitudes in some scenarios. That is, in our model, in which the population size fluctuates, we find that both cooperators and defectors can sustain themselves: both selfish risk-seekers and generous risk-averse agents can enable long-term population survival, and selfish risk-seekers can comprise the majority of the population without leading to its extinction. This out-performance of selfish risk-seekers is especially evident when social and environmental conditions are unfavourable. That is, when risk-seekers are surrounded by pure cooperators but there is a medium to high peril in engaging in risky actions (first section of the results), or when generous agents act conditionally cooperatively and the costs for risk-seeking behaviours is medium (second section of the results and *m*_*S*_ = 25%), and this remains true also when an individual cost is imposed on risk-seeking agents (last section of the results and *m*_*S*_ = 25%). Overall, results show how behaviours aligning with core characteristics of psychopathy were advantageous for agents, as long as the conditions were not extreme. Nevertheless, it is important to remember that psychopathy is a much more complex set of behaviours and we considered only a small subset in our model. While the individual benefit of selfish risk-seeking is evident, the benefit for communities is less clear. When generous agents are pure cooperators, communities with a greater proportion of selfish risk-seeking agents grow to a larger population size suggesting some advantage to the community. When generous agents behave as conditional cooperators, however, communities with a greater proportion of generous risk-averse agents reach similar sizes to populations comprising mostly selfish risk-seeking agents, leaving it unclear whether selfish risk-seeking behaviour can be beneficial not only for selfish risk-seekers themselves but also for the community.

Interestingly, in the first two models, results are qualitatively independent of the environmental conditions the population faces: whether there is an abundance or a scarcity of resources is not influential on the evolution dynamics. The lack of impact of the environmental resources provided is probably due to the fact that, when resources are scarce, the population decreases in such a way that each agent who survives can have access to an adequate amount of resources. Thus, the dynamics are mainly controlled by the maximum amount each agent can harvest and not by the overall resources available (this is further illustrated in the SI). However, when we introduce an individual cost for selfish risk-seeking agents, the scarcity or abundance of resources plays a role in the survival of the communities, leading populations to extinction in more scenarios when resources are scarce. Overall, our initial hypothesis that the adaptive side of selfish risk-seekers will prevail in environmentally scarce situations does not find support when we focus purely on environmental resources. Nonetheless, the probability of perishing when harvesting represents an aspect of environmental harshness, while the community and individual costs embody a social harshness. Thus, if we consider as source of environmental harshness the higher propensity to perish during harvesting (*m*_*S*_ ∈ {25, 50}), then selfish risk-seekers are fitter than generous risk-averse agents in such conditions. In fact, when the mortality rate was set to 25%, the probability of reproducing selfish risk-seeking offspring was always significantly higher than the probability of reproducing generous offspring, regardless of other factors. This indicates that, as long as the costs are not set too high (either by setting the mortality rate to 50% or the community cost to 100), then selfish risk-seekers perform better in situations where their survival is moderately challenged. Interestingly, when the mortality rate for selfish agents is set to 50%, then generous risk-averse agents can comprise the larger share of the population, indicating that if the conditions are too harsh for selfish risk-averse agents, then their advantage over the rest of the population decreases.

An aspect that would be of interest for future research is to allow the individual personality traits of selfishness and risk-seeking to evolve separately, thus allowing more complex behavioural combinations to arise. In this paper we consider selfish and risk-seeking attitudes as one unified behavioural profile that evolves as a single personality trait. However, an individual could express generous attitudes and at the same time be prone to engage in risky actions. By modelling each behavioural component separately, it would be possible to observe whether it is the combination of selfish and risk-seeking behaviours (that has been strongly correlated with psychopathy) that is evolutionarily adaptive or whether the evolution is driven by the dynamics of individual traits, similar to cross-sectional findings on professional success [33]. Could other combinations of the two traits invade the community? Our interpretation is that the combination of the two aspects (selfishness and risk-seeking) is what makes individuals more successful. That is because individuals who engage in risky actions gather more resources for themselves, improving their own fitness in this way. At the same time, given the heterogeneity of the community, behaving in a selfish way is the optimal solution to protect the resources gathered. In a community where individual fitness is more strongly dependent on the benefit of the population as a whole, we could expect more generous behaviours to be favoured over selfishness. Thus, depending on the society we are living in, different behaviours could arise as evolutionarily adaptive. In a society where teamwork is essential, generous behaviours will be more advantageous. On the other hand, in a structured and hierarchical society, a more selfish and self-centred attitude will yield higher rewards for the individual, making selfish and risk-seeking behaviours evolutionarily adaptive. On the other hand, if these two traits vary in their impact on populations, their effects could go in opposite directions, which would be interesting to investigate next.

Our agent-based model sheds light on the evolutionary role of a combination of traits (selfishness and risk-seeking) that is central to the psychopathy construct. Our results point towards an evolutionarily adaptive role of selfishness and risk-seeking behaviours, while also marginally supporting the adaptive theory that psychopathic traits may not be a dysfunction but rather an adaptive consequence of human evolution.

## Supporting information

S1 File (PDF)
