# Supplementary material for: Selfish risk-seeking can provide an evolutionary advantage in a conditional public goods game
Source: PLoS One. 2022 Jan 21;17(1):e0261340. doi: 10.1371/journal.pone.0261340 (PMC8782365; doi:10.1371/journal.pone.0261340)
Supplement: S1 File — (PDF) [file pone.0261340.s001.pdf]

Selfish risk-seeking can provide an evolutionary advantage in a conditional cooperator public goods game.
Supplementary Information

Contents

S1 Model **4**

S2 Environmental impact **7**

S3 Perfect transmission **9**

S4 Baseline model, no mutation **12**

S5 Additional Results **13**

S5.1 Baseline model 13

S5.2 Conditional cooperation 21

S5.3 Conditional cooperation and agent cost for selfish risk-seekers . . 30

List of Figures

S1 Environment impact,$e_{max} = 4$ 8

S2 Environment impact,$e_{max} = 4.5$ 9

S3 Perfect transmission model; population overview 10

S4 Perfect transmission model; population evolution, scarce environment 11

S5 Perfect transmission model; population evolution, abundant environment . . . 12
S6 Baseline model, no mutation; population overview . . . 13
S7 Baseline model; maximum resources comparison, scarce environment . . . 14
S8 Baseline model; maximum resources comparison, scarce environment . . . 15
S9 Baseline model; population overview . . . 16
S10 Baseline model; population evolution, scarce environment . . . 17
S11 Baseline model; population evolution, abundant environment . . . 18
S12 Baseline model; reproduction rates, scarce environment . . . 19
S13 Baseline model; reproduction parameters evolution, scarce environment . . . 20
S14 Conditional cooperation model; maximum resources comparison, scarce environment . . . 22
S15 Conditional cooperation model; maximum resources comparison, abundant environment . . . 23
S16 Conditional cooperation model; Population overview . . . 24
S17 Conditional cooperation model; population evolution, scarce environment . . . 25
S18 Conditional cooperation model; population evolution, abundant environment . . . 26
S19 Conditional cooperation model; reproduction rates evolution, scarce environment . . . 27
S20 Conditional cooperation model; reproduction parameters evolution, abundant environment . . . 28
S21 Conditional cooperation model; reproduction parameters evolution, scarce environment . . . 29
S22 Conditional cooperation and individual cost model; maximum resources comparison, scarce environment . . . 30
S23 Conditional cooperation and individual cost model; maximum resources comparison, abundant environment . . . 31
S24 Conditional cooperation and individual cost model; cost sensibility, abundant environment . . . 32
S25 Conditional cooperation and individual cost model; cost sensibility, scarce environment . . . 33

S26 Conditional cooperation and individual cost model; population evolution, scarce environment 34
S27 Conditional cooperation and individual cost model;population evolution, abundant environment 35
S28 Conditional cooperation and individual cost model; reproduction rates evolution, abundant environment 36
S29 Conditional cooperation and individual cost model; reproduction parameters evolution, scarce environment 37
S30 Conditional cooperation and individual cost model; reproduction parameters evolution, abundant environment 38

S1 Model

The initial population is composed of N_0 agents, whereof 10% are selfish risk-seekers.

Harvesting

First, agents gather resources from the environment (harvesting phase). The environment provides a fixed amount of resources per generation $e_{tot} = e * N_0$, independent of the population size. Each agent can harvest an equal proportion of the total amount $e_t = e_{tot}/N_t$, which varies according to the number of agents in the population. Risk-seekers harvest 80% of the available resources, while the risk-averse gather 50% of the resources offered by the environment. The amount gathered by agent i in generation t is given by

$$r_{i,t} = e_t h_{p_i}, \tag{1}$$

where h_{p_i} is the harvesting rate of phenotype p_i. Note that each agent cannot harvest more than $e_{max} = 4.5$ units of resources per generation. This is implemented to limit the surplus of resources each agent can harvest in one generation During this first step, agents perish with a probability m_{p_i} as a consequence of engaging in the risky action of harvesting, where p_i defines the phenotype of agent i (mortality phase). Since risk-averse agents do not engage in risky actions, they are not affected by the mortality phase ($m_G = 0$). Thus, the initial population N_0 evolves at time t to

$$N_t = \tilde{N}_t - (m_S * S_t + m_G * G_t), \tag{2}$$

where $\tilde{N}_t$ is the population size at time t before the mortality occurs, and S_t/G_t is the proportion of risk-seekers/risk-averse in the population at generation t. After the harvesting phase, agent i has an amount of resources $r_{i,t}$ that depends on both the environmental offer e_t and their phenotype p_i.

Public Goods Game

Second, they participate in a Public Goods Game (PGG) in which they contribute a portion of their resources to the communal pot:

$$\tilde{C}_t = \sum_{j=0}^{N_t} r_{j,t} c_{p_j}, \tag{3}$$

where c_{p_j} represents the percentage of the total resources agent i contributes to the public good, while $\tilde{C}_t$ represents the total amount of contributions. The total amount in the pot will be multiplied by a constant factor ρ that represents the return on investing in the community. Generous risk-averse agents are modelled to contribute 100% of their resources (at a later stage they are modelled as conditional cooperators), while selfish risk-seekers contribute only 20%.

After having gathered all the agents' contributions and before redistribution, the community pays a cost proportional to the number of selfish risk-seeking agents. This represents the cost that anti-social, selfish and risk-seeking agents impose on the community through their anti-social behaviours, for example by breaking the rules or disrupting the community. The remaining communal pot after the payment is given by:

$$C_t = \tilde{C}_t(1 - \lambda * S_t), \tag{4}$$

where λ describes the percentage of resources that the community would have to pay if the population were entirely composed of selfish-risk seekers. The communal pot is then redistributed equally among all agents. Each agent's fitness $f_{i,t}$ is composed of the resources they gathered and their return from the PGG:

$$f_{i,t} = \frac{C_t \rho}{N_t} + r_{i,t}(1 - c_{p_i}). \tag{5}$$

Reproduction

Thirdly, each agent reproduces proportionally to their fitness after the PGG phase. If they did not gather enough resources to meet the survival threshold, they perish without offspring. That is, there is a minimum of resources each agent needs to acquire in order to reproduce, which allows agents to produce an offspring. The number of offspring an agent produces is given by:

$$n_{i,t} = \begin{cases} 0, & \text{if } f_{i,t} \leq s \\ \lceil \frac{o}{M-s}(f_{i,t} - s) \rceil, & \text{if } s < \ f_{i,t} < M \\ o, & \text{if } f_{i,t} \geq M \end{cases} \tag{6}$$

where $M = s * o + s$ is the necessary fitness for an agent to reproduce the maximum o offspring. In order to avoid an infeasible number of offspring, we

fixed an upper limit of o offspring per agent. This represents a natural limit of offspring an agent can produce in a lifetime.

The usual reproduction mechanism in an evolutionary model is characterised by $\varphi_{G,G} = \varphi_{S,S} = 1$, that is, offspring inherit their phenotype directly from their parents. However, in our model, the offspring's phenotype is determined by a reproduction mechanism which takes into account the phenotype expressed by the parent, but it might not be the same. The reproduction rates of each agent are composed of two features, a part ϕ common across the population and a part γ or ω, specific to the parental phenotype:

$$\varphi_{G,G} = 1 - \phi + \omega, \tag{7}$$

$$\varphi_{G,S} = \phi - \omega, \tag{8}$$

$$\varphi_{S,S} = \phi + \gamma, \tag{9}$$

$$\varphi_{S,G} = 1 - \phi - \gamma. \tag{10}$$

$$0 \leq \varphi_{G,G}, \varphi_{G,S}, \varphi_{S,G}, \varphi_{S,S} \leq 1 \tag{11}$$

where $\varphi_{pi,pj}$ represents the probability of a p_i parent reproducing a p_j offspring; ϕ represents the population baseline propensity of each agent to reproduce selfish risk-seeking agents; γ $(0 \leq \gamma \leq 1 - \phi)$ embodies the effect of having a selfish risk-seeking parent in the development of personality traits; while ω $(0 \leq \omega \leq \phi)$ embodies the effect of having a generous risk-averse parent in the development of personality traits. γ represents an increased tendency to develop selfish risk-seeking personality traits due to factors such as the lack of time invested in parenting, a potentially scarcer environment experienced during childhood and the possibility of inheriting a genetic tendency towards selfish risk-seeking traits from the parent. On the other hand, ω represents a decreased tendency to develop selfish risk-seeking traits, due to both genetic and environmental factors such as more benevolent surroundings when growing up. That is, a selfish risk seeker will reproduce a selfish offspring with probability $\phi + \gamma$ and a generous risk-averse agent will reproduce a generous offspring with probability $1-(\phi-\omega)$. The reason why we modified the common reproduction mechanism is due to the fact that selfish risk-seeking traits are usually less common in the general population (thus ϕ is small) and individuals might exhibit such traits as a consequence of environmental cues (ω, ϕ) [1]

[1]Hyde et al. (2016) looked at the heritable pathways to early callous-unemotional be-

Mutation

Lastly, a mutation in the reproduction probabilities is introduced. At each generation, the reproduction probabilities are subject to a small mutation (mutation phase). Mutations $\eta_{\phi,t}$, $\eta_{\gamma,t}$ and $\eta_{\omega,t}$ for ϕ, γ and ω are drawn from a $\mathcal{N}(0, \sigma^2)$. Thus, the reproduction probabilities evolve over generations as follows:

$$\varphi_{G,G,t} = 1 - \phi_t + \omega_t = 1 - \phi_{t-1} - \eta_{\phi,t} + \omega_{t-1} + \eta_{\omega,t}, \tag{12}$$

$$\varphi_{G,S,t} = \phi_t - \omega_t = \phi_{t-1} + \eta_{\phi,t} - \omega_{t-1} - \eta_{\omega,t}, \tag{13}$$

$$\varphi_{S,S,t} = \phi_t + \gamma_t = \phi_{t-1} + \eta_{\phi,t} + \gamma_{t-1} + \eta_{\gamma,t}, \tag{14}$$

$$\varphi_{S,G,t} = 1 - \phi_t - \gamma_t = 1 - \phi_{t-1} + \eta_{\phi,t} - \gamma_{t-1} - \eta_{\gamma,t}, \tag{15}$$

where we enforce $\varphi_{G,G,t}, \varphi_{G,S,t}, \varphi_{S,S,t}, \varphi_{S,G,t} \in [0, 1]$ by defining an upper and lower limit to the mutations if necessary. Note that this has not been necessary in any of the conditions reported in the results.

S2 Environmental impact

We investigate the effects of different environmental settings in a basic scenario: agents always reproduce offspring that carry their own phenotype, that is $\varphi_{G,G} = \varphi_{S,S} = 1$. This is the usual reproduction mechanism in evolutionary games. While we go on to use the reproduction mechanism described above that more closely captures features of the inheritance of personality traits, we summarise here what happens with this simple inheritance mechanism. We define an abundant scenario when population survives and grows in most conditions. On the other hand, a scarce environment is defined as one where the population still survives in most scenarios but its final equilibrium size is less or equal to the initial size. Figure S2 reports the population size at equilibrium after 10^4 generations. The initial population was $N_0 = 250$ in all cases, and the maximum resources an agent can gather in each generation was $e_{max} = 4.5$.

haviours, which can be considered the precursors of psychopathy [1]. They sampled 561 families that adopted children whose biological mothers showed severe antisocial behaviours. Their aim was to investigate the heritability of such traits despite rare contact between the children and their biological mothers. Results showed that children inherited callous and unemotional behaviours from their biological mothers, but positive reinforcement of the adoptive mothers buffered such behaviours. These results are in line with previous findings that 69% of the variance in latent psychopathic traits are explained by genetic factors, while 31% of it seems to be driven by environmental conditions [2].

The environmental condition that satisfies our definition of a scarce environment corresponds to $e = 1.2$. In this case, the population survives in five scenarios out of nine and its size is usually lower than the initial one ($N_0 = 250$). This suggests an environmental condition that allows the population to survive in most cases but not to flourish over time. On the other hand, for the abundant environment we chose $e = 4.5$, where the population survives in most cases (apart from when $m_S = 0\%$ and $\lambda = 0, 100$) and the equilibrium population is greater than the initial one in most of the scenarios. This makes the environment $e = 4.5$ favourable for the growth and the proliferation of the population. This choice of abundant environment also motivated the choice of $e_{max} = 4.5$. Given the closeness in performance of $e = 4$ and $e = 4.5$, we provide the evolution of the population also for $e = 4, e_{max} = 4$ for each of the models investigated.

Figure S1: Final population size depending on the resources available per generation ($e_{tot} = e * N_0$), with $e_{max} = 4$.

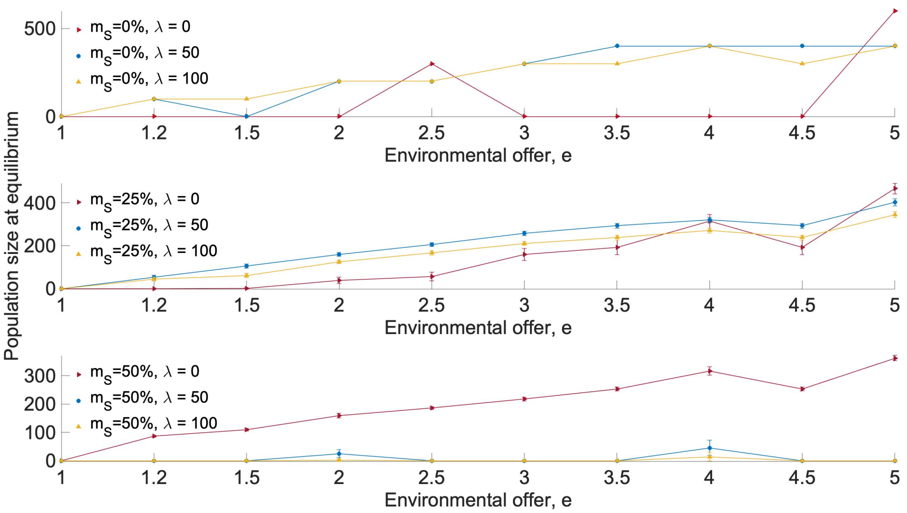

Figure S2: Final population size depending on the resources available per generation ($e_{tot} = e * N_0$), with $e_{max} = 4.5$.

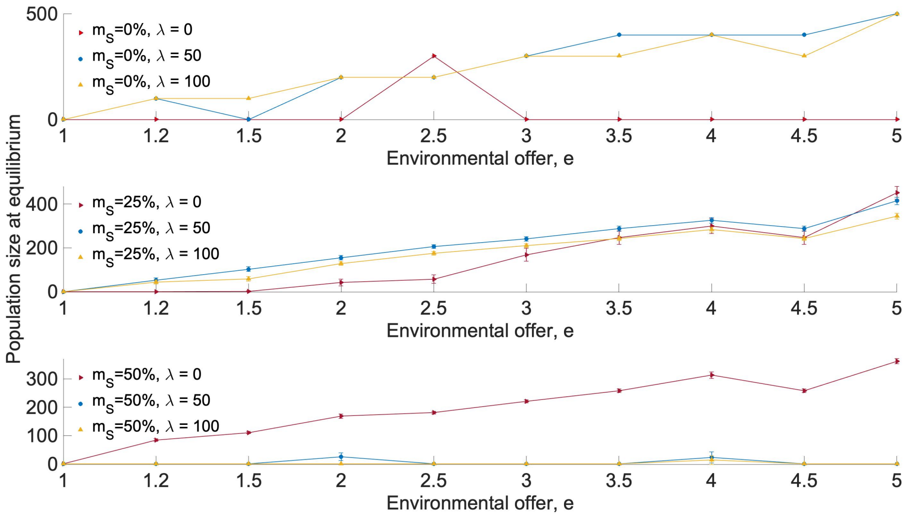

S3 Perfect transmission

First, we present the results for a simple model in which agents always reproduce offspring that carry their own phenotype, that is $\varphi_{G,G} = \varphi_{S,S} = 1$. When agents pass on their phenotypes to their offspring, selfish risk-seeking agents invade the community in most scenarios (only exception, high mortality rate and abundant resources, see Figure S3). Interestingly, if resources are abundant and the cost for risky actions is high ($m_S = 50\%$), then generous risk-averse agents either invade the community (if the community cost is either 50 or 100) or comprise the majority of the population.

Figure S3: Equilibrium state for populations in (a) a scarce ($e = 1.2$) and (b) an abundant ($e = 4.5$) environment. The color shade represents the population size at equilibrium, while the percentages represent the proportion of generous risk-averse agents in the population at equilibrium. White tiles indicate that the population reached extinction by the 10^4 generation. Phenotypes are transmitted perfectly, meaning that selfish risk-seeking agents reproduce selfish risk-seeking offspring and vice-versa.

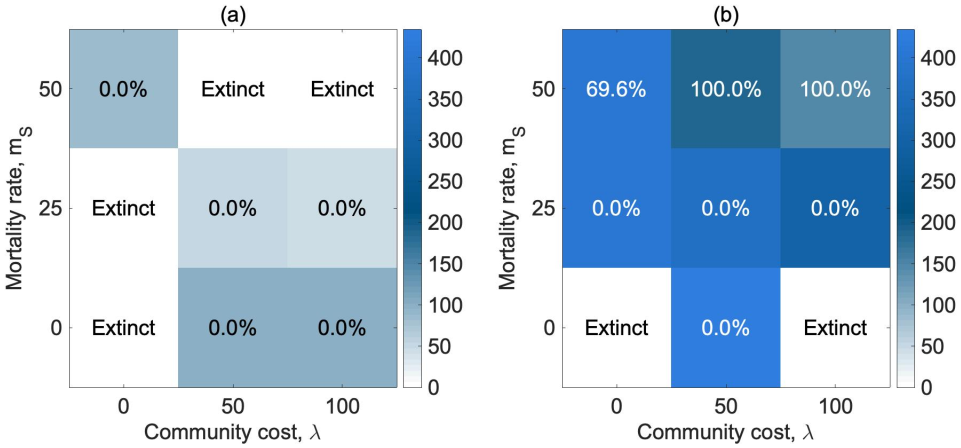

Figure S4: Evolution of the population over generations in a scarce environment ($e = 1.2$) when phenotypes are transmitted perfectly, meaning that selfish risk-seeking agents reproduce selfish risk-seeking offspring and vice-versa.
Red area: Selfish risk-seeking agents, **Yellow** area: Generous risk-averse agents.

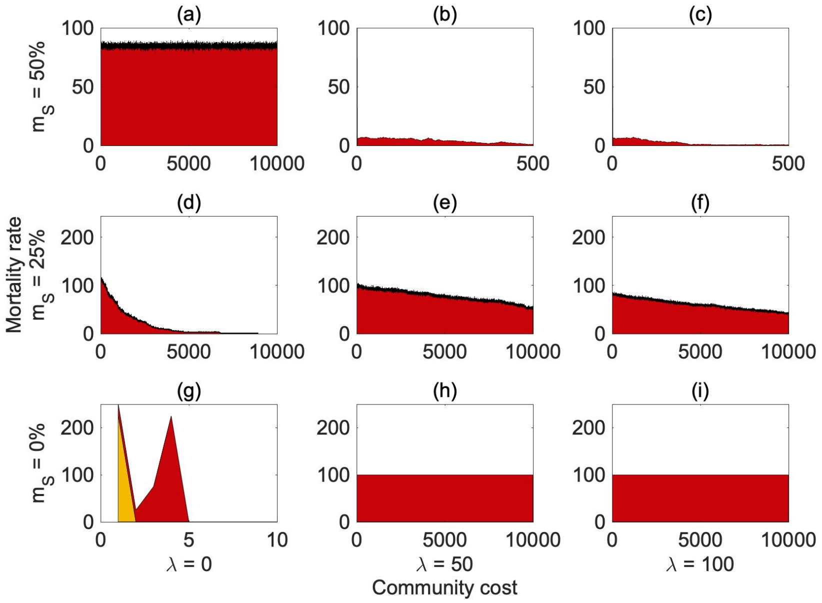

Figure S5: Evolution of the population over generations in an abundant environment ($e = 4.5$) when phenotypes are transmitted perfectly, meaning that selfish risk-seeking agents reproduce selfish risk-seeking offspring and vice-versa.
Red area: Selfish risk-seeking agents, **Yellow** area: Generous risk-averse agents.

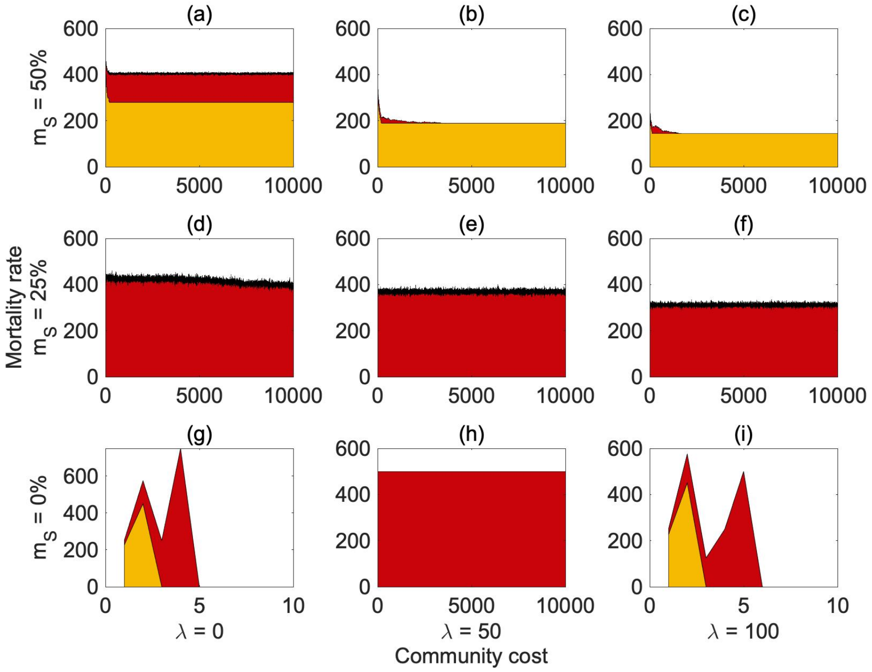

S4 Baseline model, no mutation

Second, we show the results for the baseline model (as presented in the main text) in which no mutation is introduced, meaning that generous agents always have a 91% chance of reproducing generous risk-averse offspring, while selfish agents always have an 11% chance of reproducing selfish risk-seeking offspring. Populations maintain similar proportions of selfish risk-seeking and generous risk-averse agent to those initialised at the beginning of the model (90% generous risk-averse/10% selfish risk-seeking), see Figure S6. In both environments, the population size grows to reach similar values to those reported in Figure S4/5(a) for scarce/abundant environments. Since agents have a high probability of reproducing generous offspring (91% for generous-risk averse agents and

89% for selfish risk-seekers), most offspring produced by selfish parents also exhibit the generous phenotype, thus allowing the population to reach stability. On the other hand, when mutation is allowed, the advantage provided by selfish risk-seekers become evident and more and more selfish agents are reproduced over generations

Figure S6: Equilibrium state for populations in (a) a scarce ($e = 1.2$) and (b) an abundant ($e = 4.5$) environment when no mutation is implemented. The color shade represents the population size at equilibrium, while the percentages represent the proportion of generous risk-averse agents in the population at equilibrium.

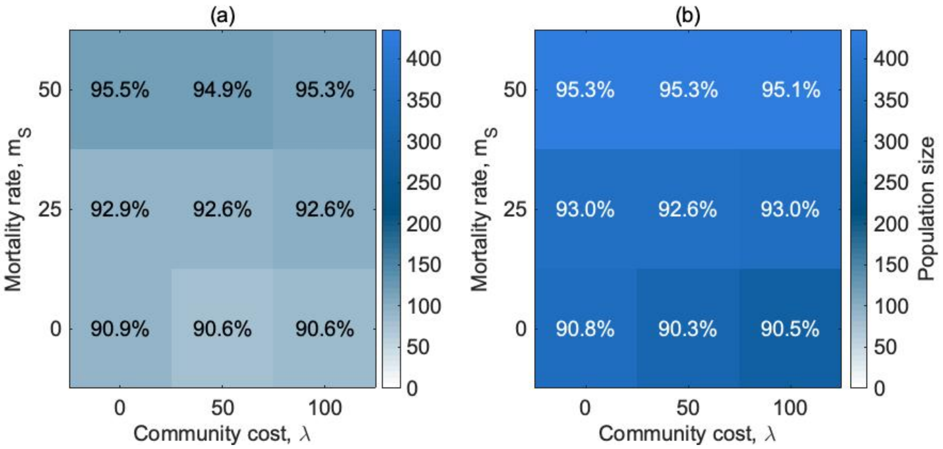

S5 Additional Results

S5.1 Baseline model

Here, we present the equilibrium size of the population when $e_{max} = 4$ and $e_{max} = 4.5$ for the model presented in section 3.1. Figures S7 and S8 show the equilibrium population size as well as the percentage of selfish agents when changing the maximum amount of resources each agent can gather, for both an abundant ($e = 4/4.5$) and a scarce ($e = 1.2$) environment. Results are quite consistent across the two conditions. The only difference is that, when $m_S = 50\%$ and $\lambda = 50$, the population goes extinct if agents can gather a maximum of 4 units of resources per generation, but not if they can gather 4.5 units of resources (both when the total resources are scarce or abundant).

Figure S7: Final population size and composition when changing the maximum amount of resources an agent can gather e_{max} from 4 to 4.5. The resources provided are scarce (e=1.2).

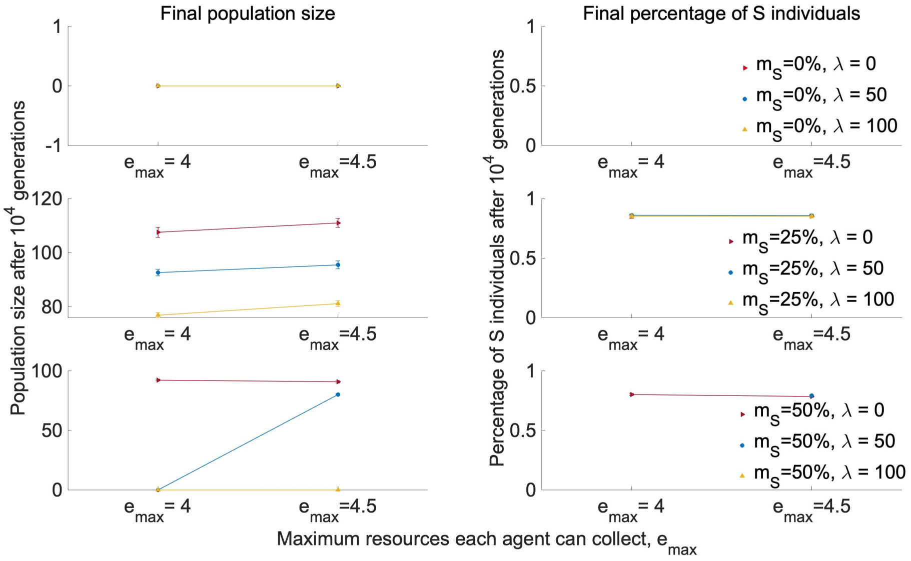

Figure S8: Final population size and composition when changing the maximum amount of resources an agent can gather e_{max} = 4 to 4.5. The resources provided are abundant (e=4/4.5).

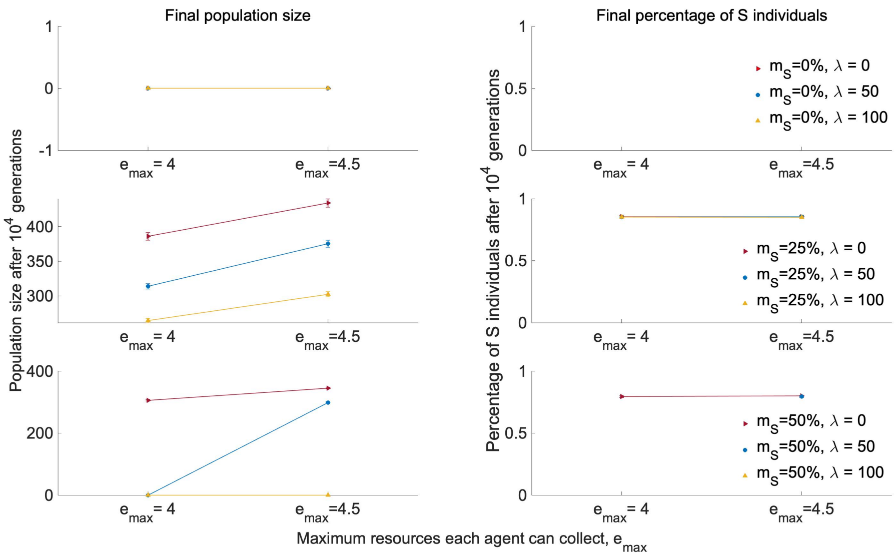

Results show the evolution of the populations over the 10^4 generations for the model described in section 3.1. Figure S9 illustrates the overview of the population size and composition in both scarce and abundant environments. Figure S10 shows the evolution over generations of the population in a scarce environment, while Figure S11 in an abundant environment. Also, Figure S12 and Figure S13 report the evolution of the reproduction parameters in a scarce environment.

Figure S9: Equilibrium state for populations in (a) a scarce ($e = 1.2$) and (b) an abundant ($e = 4.5$) environment. The color shade represents the population size at equilibrium, while the percentages represent the proportion of generous risk-averse agents in the population at equilibrium. White tiles indicate that the population reached extinction by the 10^4 generation.

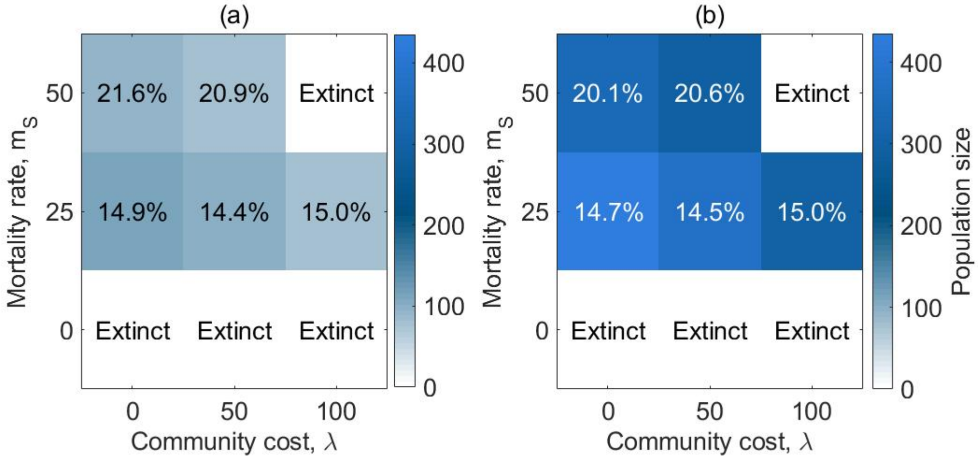

Figure S10: Evolution of the population over generations in a scarce environment ($e = 1.2$). **Red** area: Selfish risk-seeking agents, **Yellow** area: Generous risk-averse agents.

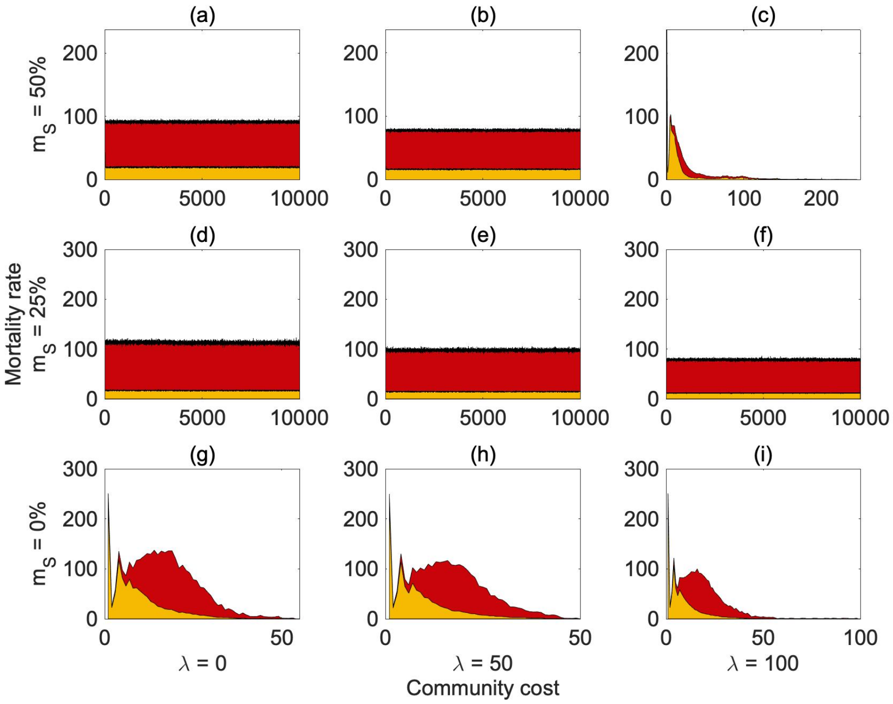

Figure S11: Evolution of the population over generations in an abundant environment ($e = 4.5$). **Red** area: Selfish risk-seeking agents, **Yellow** area: Generous risk-averse agents.

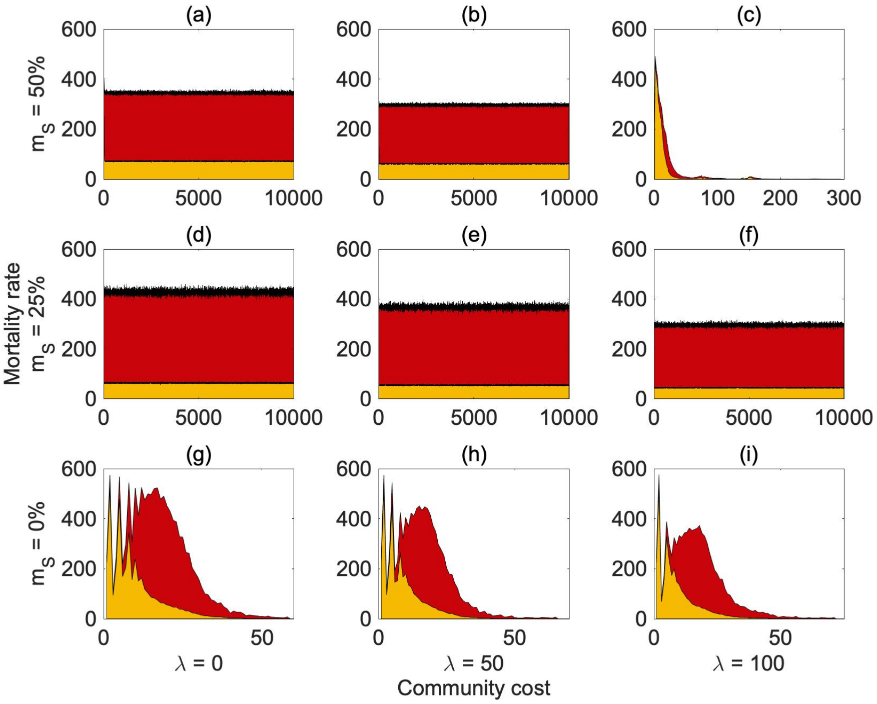

Figure S12: Evolution of the combined reproduction rates $\varphi_{S,S}, \varphi_{G,G}$ over generations in a scarce environment. **Red** line: $\varphi_{S,S}$, **Yellow** line: $\varphi_{G,G}$.
$\varphi_{G,G} = 1(0)/\varphi_{S,S} = 1(0)$ means that a generous risk-averse/selfish risk-seeking agent has a 100(0)% chance of reproducing a generous risk-averse/selfish risk-seeking offspring.

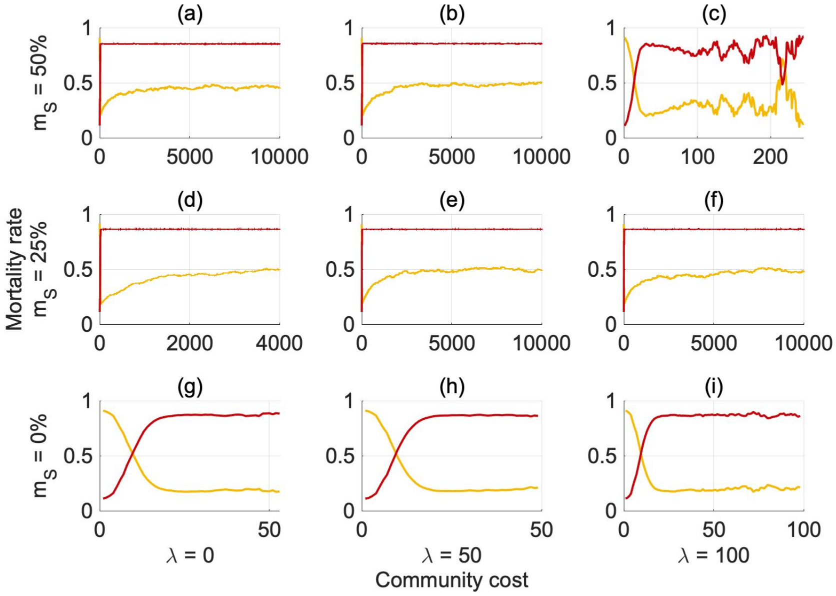

Figure S13: Evolution of the separate parameters (ω, γ and ϕ) regulating the reproduction mechanism over generations in a scarce environment ($e = 1.2$).

Red line: γ, contribution of selfish risk-seeking parent to the probability of reproducing selfish risk-seeking offspring,

Yellow line: ω, contribution of generous risk-averse parent to the probability of reproducing generous risk-averse offspring,

Black line: ϕ, probability that agents will reproduce selfish risk-seeking offspring, independent of their own phenotype.

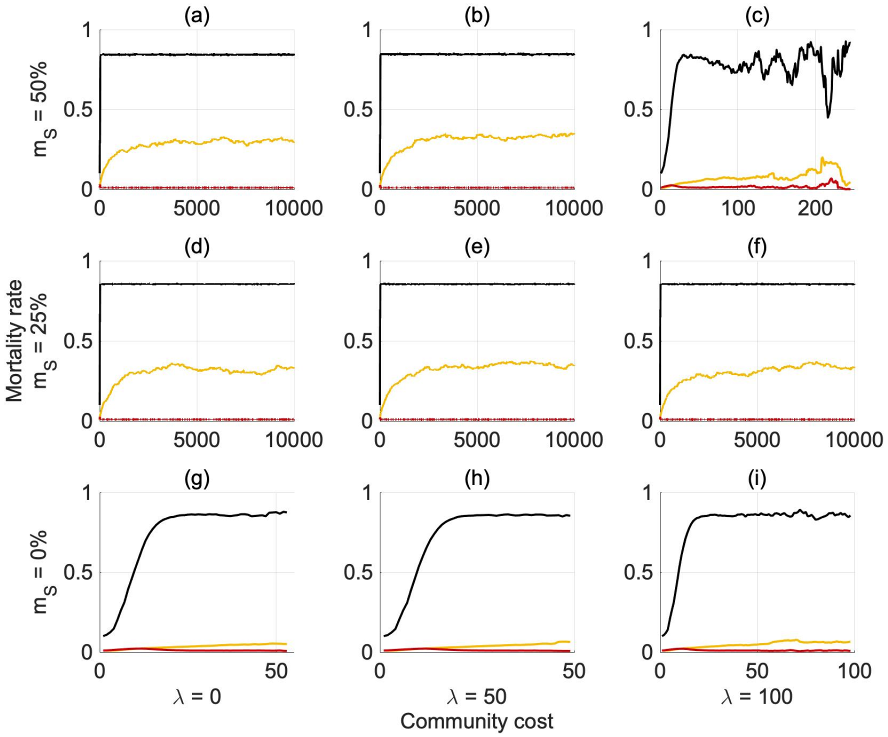

S5.2 Conditional cooperation

We present the equilibrium size of the population when $e_{max} = 4$ and $e_{max} = 4.5$ for the model presented in section 3.2. Figures S14 and S15 show the equilibrium population size as well as the percentage of selfish agents when changing the maximum amount of resources each agent can gather, for both an abundant ($e = 4/4.5$) and a scarce ($e = 1.2$) environment. Results are consistent across the two conditions when no mortality rate is introduced. If the mortality rate for risk-seekers is set to 25% and $\lambda = 0$, then the population perishes for $e = 1.2, e_{max} = 4.5$ while it reaches a stable, non-zero equilibrium for $e = 1.2, e_{max} = 4$ (if the environmental resources are abundant, the population does not perish but it reaches a very low size). On the other hand, if the mortality rate for risk-seekers is increased to 50%, the communities reach extinction for $e = 1.2/4, e_{max} = 4$ but not when $e_{max} = 4.5$, except when $e = 1.2, \lambda = 100$ in which case both communities perish. These results suggest that the maximum amount of resources each agent can harvest, and not the actual amount of resources provided by the environment, is the component that drives populations to survive or go extinct. Note that, despite the different population sizes reached, the composition of the populations remains unchanged by e_{max}. Thus, although e_{max} regulates the population size at equilibrium, it does not impact the evolution of the population composition, and therefore has no impact on the evolution of the reproduction rates.

Figure S14: Final population size and composition when changing the maximum amount of resources an agent can gather e_{max} from 4 to 4.5, when generous risk-averse agents act as conditional cooperators. The resources provided are scarce (e=1.2).

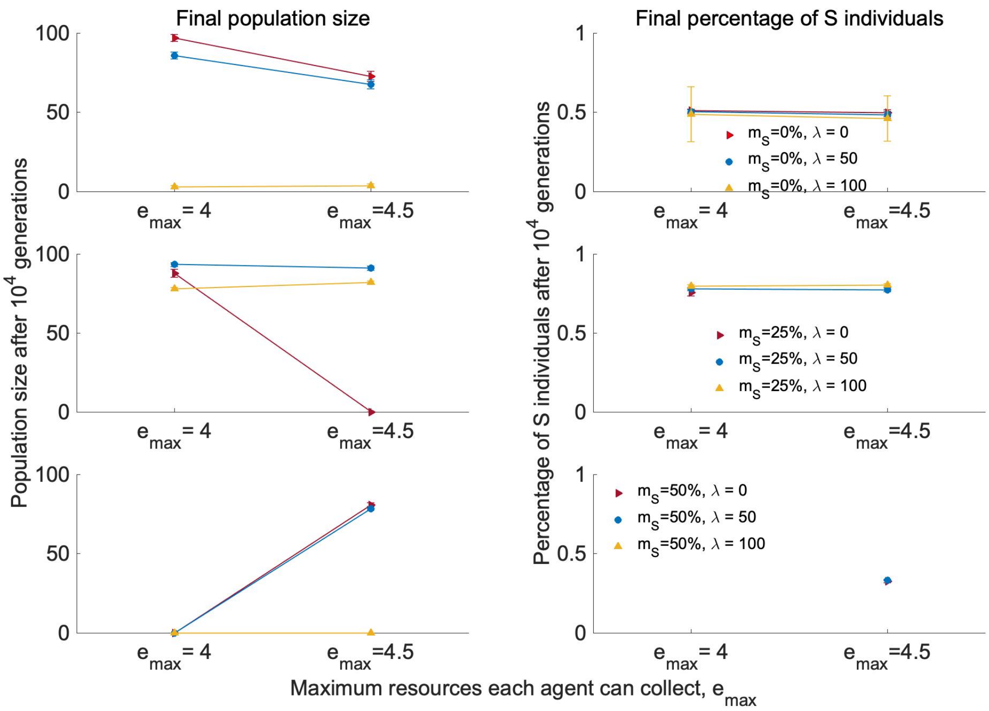

Figure S15: Final population size and composition when changing the maximum amount of resources an agent can gather e_{max} from 4 to 4.5, when generous risk-averse agents act as conditional cooperators. The resources provided are abundant (e=4/4.5).

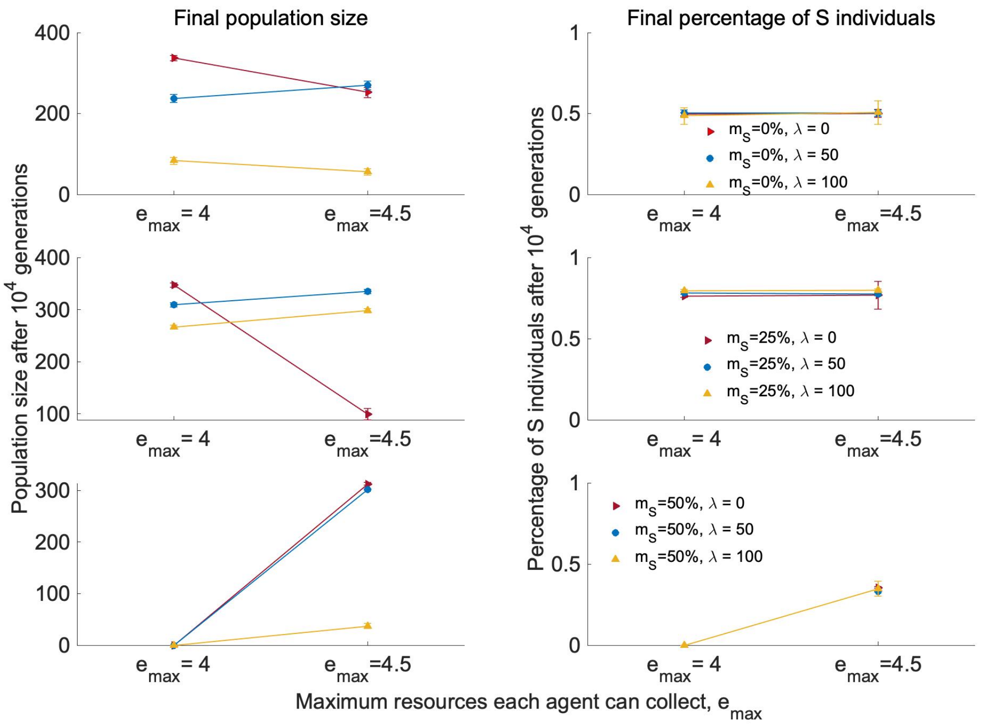

Results hereafter show the evolution of the populations over the 10^4 generations, when generous risk-averse agents act as conditional cooperators as in section 3.2. Once again, Figure S16 illustrates the overview of the population size and composition in both abundant and scarce environments. Figure S17 shows the evolution over generations of the population in a scarce environment, while Figure S18 in an abundant environment. Also, Figure S19 report the evolution of the reproduction rates in a scarce environment. Figures S20, S21 report the evolution of the reproduction parameters in both an abundant and a scarce environment.

Figure S16: Equilibrium state for populations in (a) a scarce ($e = 1.2$) and (b) an abundant ($e = 4.5$) environment, when generous risk-averse agents act as conditional cooperators. The color shade represents the population size at equilibrium, while the percentages represent the proportion of generous risk-averse agents in the population at equilibrium. White tiles indicate that the population reached extinction by the 10^4 generation.

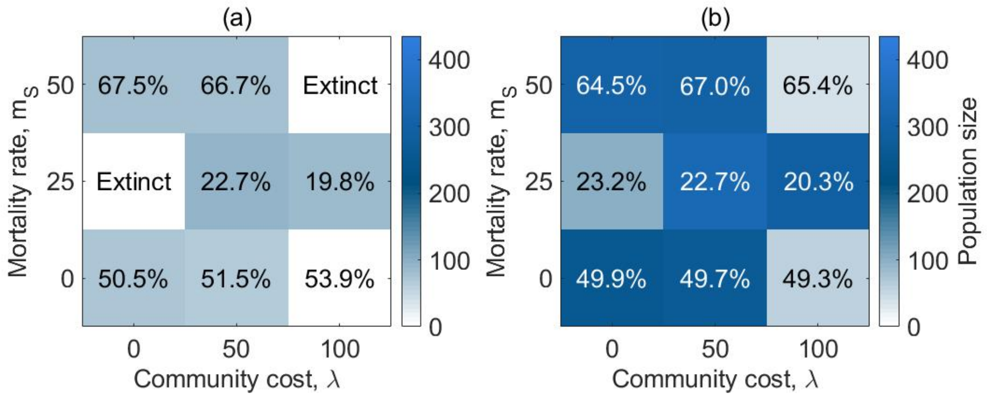

Figure S17: Evolution of the population over generations in a scarce environment ($e = 1.2$), when generous risk-averse agents act as conditional cooperators.
Red area: Selfish risk-seeking agents, **Yellow** area: Generous risk-averse agents.

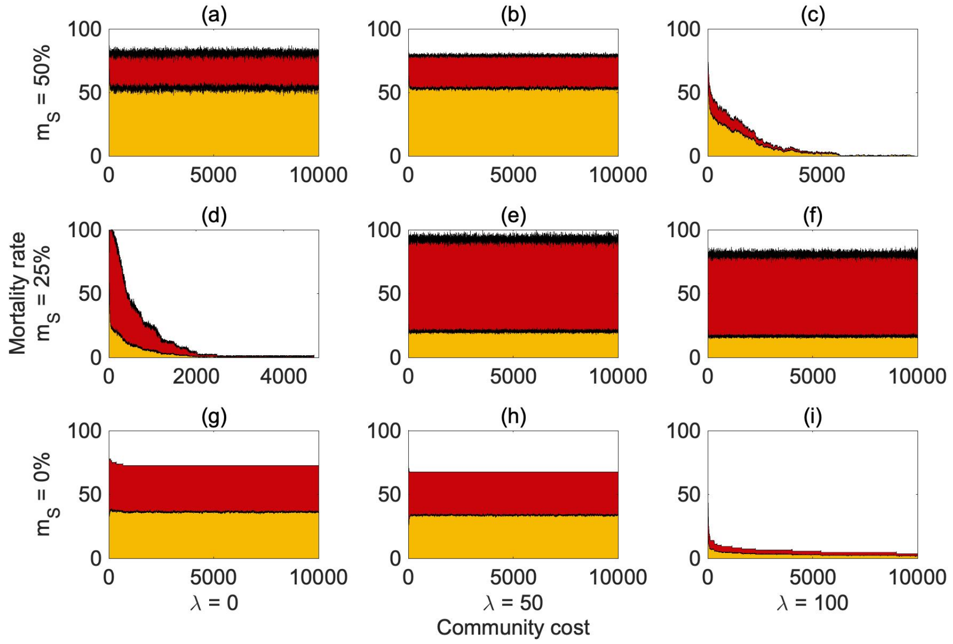

Figure S18: Evolution of the population over generations in an abundant environment ($e = 4.5$), when generous risk-averse agents act as conditional cooperators.
Red area: Selfish risk-seeking agents, **Yellow** area: Generous risk-averse agents.

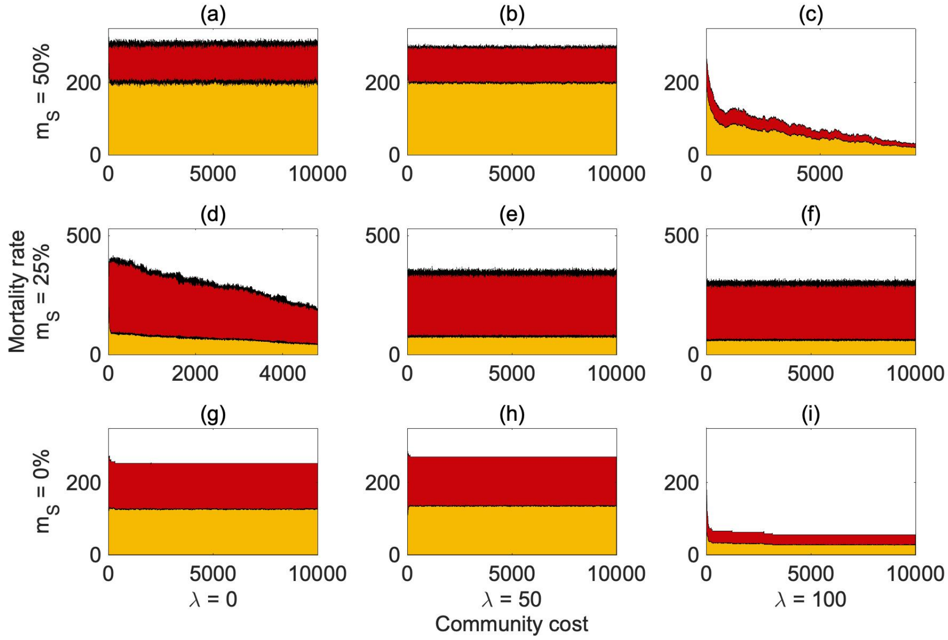

Figure S19: Evolution of the combined reproduction rates $\varphi_{S,S}, \varphi_{G,G}$ over generations in a scarce environment ($e = 1.2$), when generous agents behave as conditional cooperators. **Red** line: $\varphi_{S,S}$, **Yellow** line: $\varphi_{G,G}$.
$\varphi_{G,G} = 1(0)/\varphi_{S,S} = 1(0)$ means that a generous risk-averse/selfish risk-seeking agent has a 100(0)% chance of reproducing a generous risk-averse/selfish risk-seeking offspring.

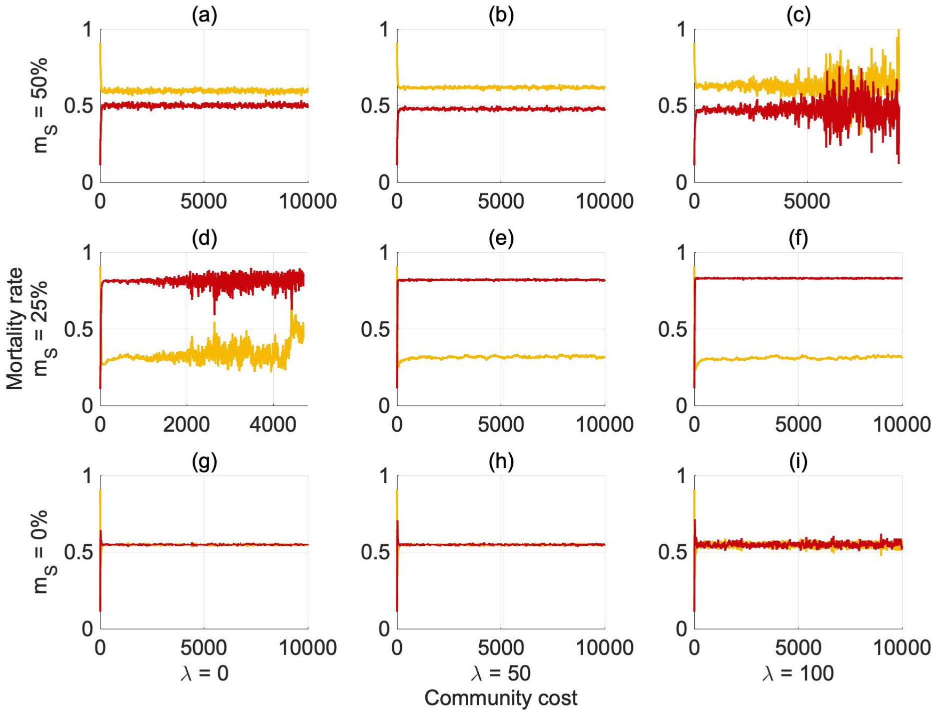

Figure S20: Evolution of the separate parameters (ω, γ and ϕ) regulating the reproduction mechanism (eq. 7-11) over generations in an abundant environment ($e = 4.5$), when generous agents behave as conditional cooperators.

Red line: γ, contribution of selfish risk-seeking parent to the probability of reproducing selfish risk-seeking offspring,

Yellow line: ω, contribution of generous risk-averse parent to the probability of reproducing generous risk-averse offspring,

Black line: ϕ, probability that agents will reproduce selfish risk-seeking offspring, independent of their own phenotype.

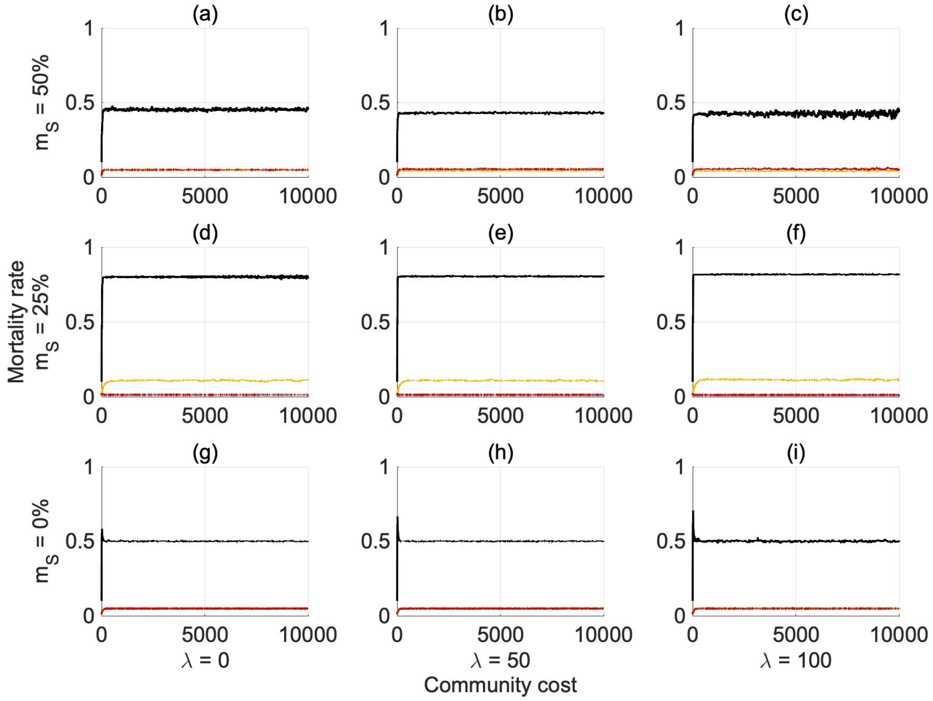

Figure S21: Evolution of the separate parameters (ω, γ and ϕ) regulating the reproduction mechanism (eq. 7-11) over generations in a scarce environment ($e = 1.2$), when generous agents behave as conditional cooperators.

Red line: γ, contribution of selfish risk-seeking parent to the probability of reproducing selfish risk-seeking offspring,

Yellow line: ω, contribution of generous risk-averse parent to the probability of reproducing generous risk-averse offspring,

Black line: ϕ, probability that agents will reproduce selfish risk-seeking offspring, independent of their own phenotype.

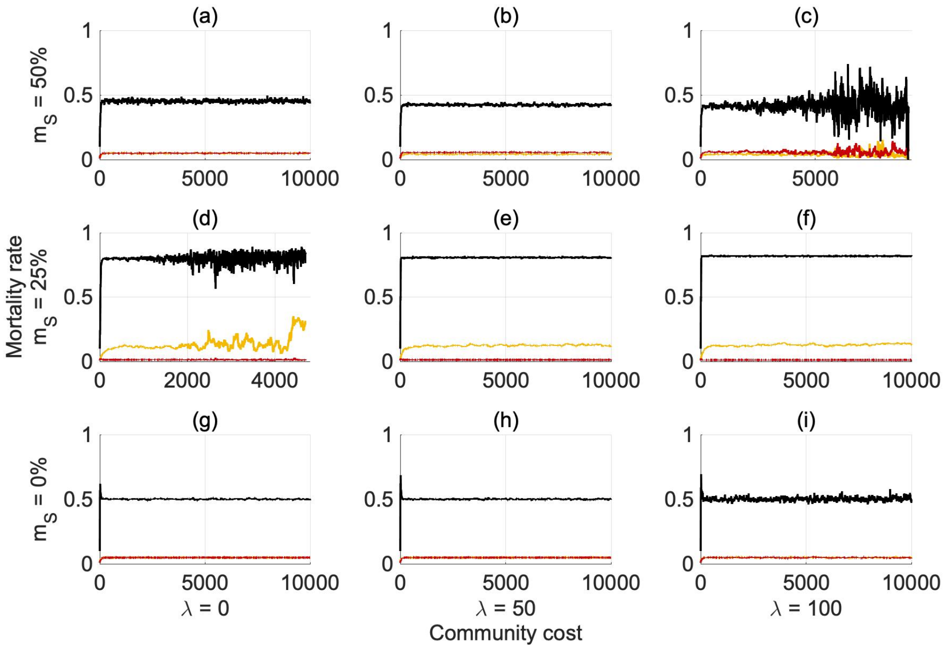

S5.3 Conditional cooperation and agent cost for selfish risk-seekers

We first present the equilibrium size of the population when $e_{max} = 4$ and $e_{max} = 4.5$ for the model presented in section 3.3 (agent cost $\alpha = 0.1$). Figures S22 and S23 show the equilibrium population size as well as the percentage of selfish agents when changing the maximum amount of resources each agent can gather, for both an abundant ($e = 4/4.5$) and a scarce ($e = 1.2$) environment. Similar to the model where only conditional cooperation is implemented, the main difference is in the survival of the populations when mortality rate is set to either 25% (communities reach extinction more often when $e_{max} = 4.5$ for $\lambda = 0$) or 50% (communities reach extinction more often when $e_{max} = 4$ for $\lambda = 0$).

Figure S22: Final population size and composition when changing the maximum amount of resources an agent can gather e_{max} from 4 to 4.5, when G agents act as conditional cooperators and S agents bear an agent cost. The resources provided are scarce (e=1.2).

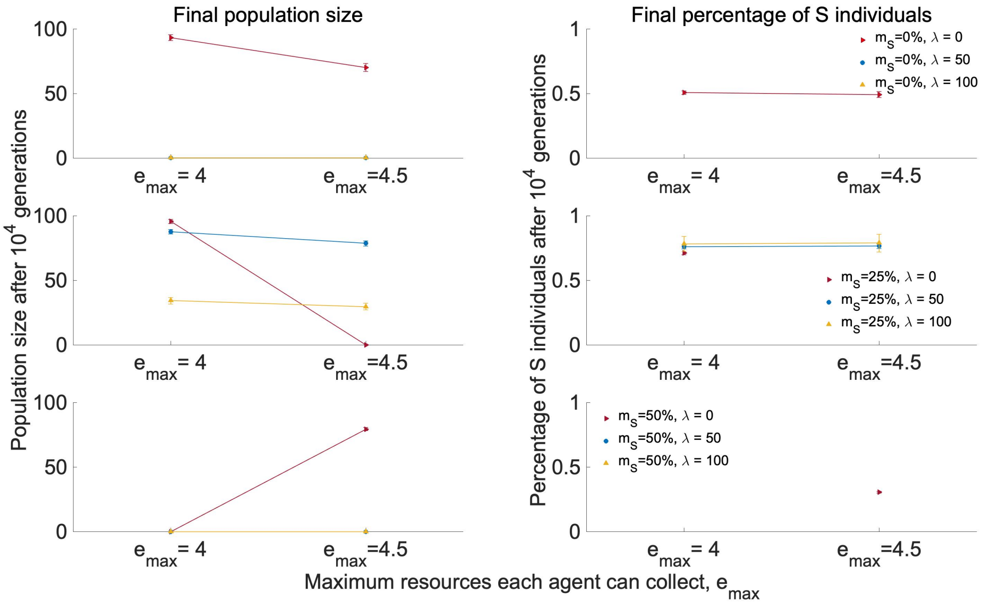

Figure S23: Final population size and composition when changing the maximum amount of resources an agent can gather e_{max} from 4 to 4.5, when G agents act as conditional cooperators and S agents bear an agent cost. The resources provided are abundant (e=4/4.5).

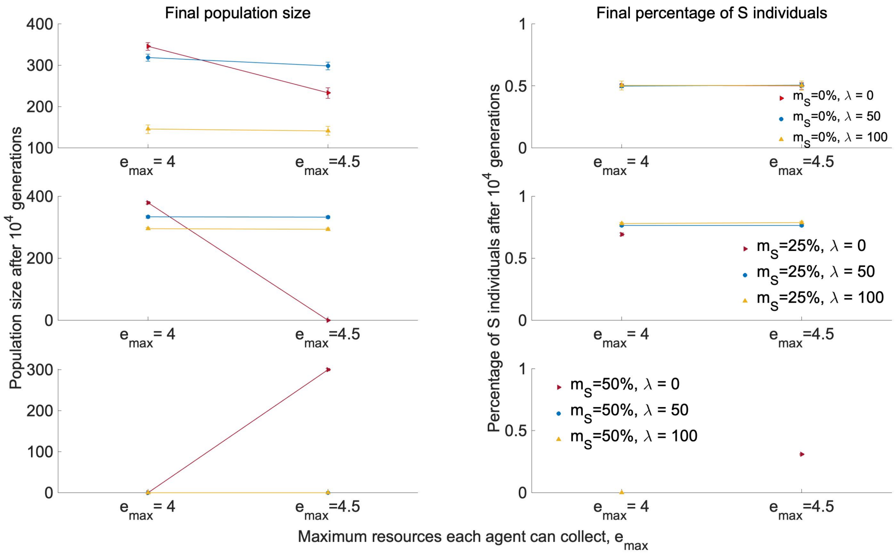

Figures S24, S25 show the evolution of the population size for the model described in section 3.3 when the individual cost for risk-seekers varies between 0 and 1 ($\alpha \in \{0.1, 0.15, 0.25, 0.5, 1\}$).

Figure S24: Final population size depending on the agent cost imposed on selfish risk-seeking agents, when the environment is abundant ($e = 4.5$) and G agents act as conditional cooperators and S agents bear an agent cost.

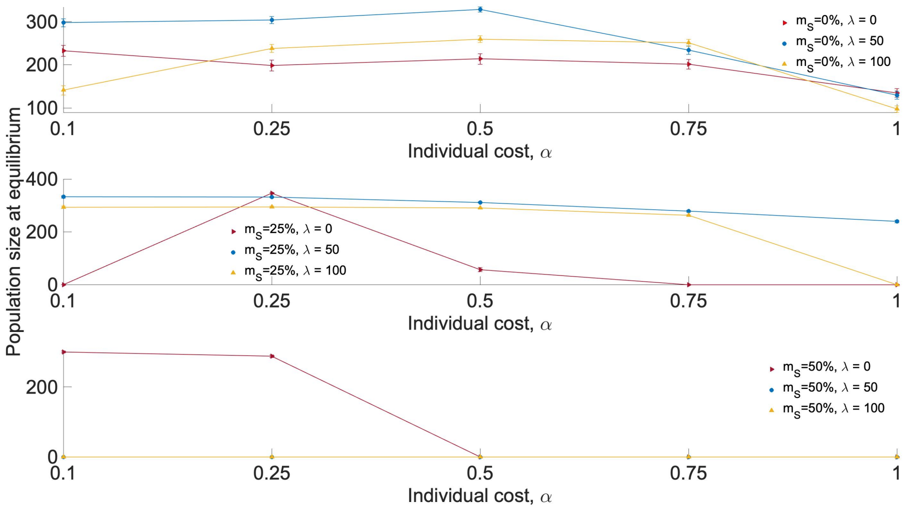

Figure S25: Final population size depending on the agent cost imposed on selfish risk-seeking agents, when the environment is scarce ($e = 1.2$) and G agents act as conditional cooperators and S agents bear an agent cost.

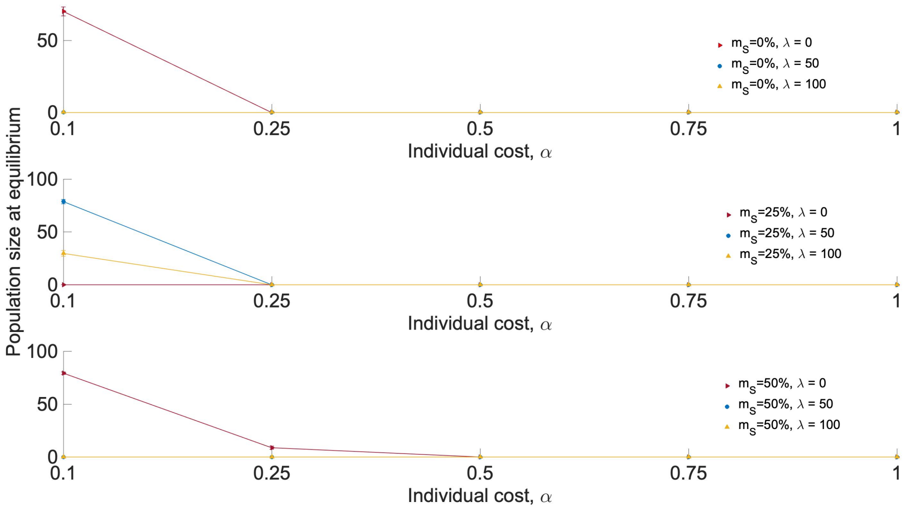

As Figures S25 and S24 show, population size changes according to the cost imposed on selfish risk-seekers. If the resources are scarce, communities go extinct for agent costs greater than 0.15. On the other hand, if the resources are abundant, communities survive in several conditions, even for high costs (α =0.5). However, independent of the environmental offer, most populations go extinct before the 10^4 generation when the cost is equal to the survival amount per agent ($\alpha = 1$).

Hereafter, results show the evolution of the populations over the 10^4 generations, when generous risk-averse agents act as conditional cooperators and an agent cost is imposed on selfish risk-seekers. Figure S26 shows the evolution over generations of the population in a scarce environment, while Figure S27 in an abundant environment. Figure S28 illustrates the evolution of the reproduction rates in a scarce environment. Moreover, Figures S29, S30 show the evolution of ω, γ and ϕ over generations in a scarce and an abundant environment respectively.

Figure S26: Evolution of the population over generations in a scarce environment ($e = 1.2.2$) when G agents act as conditional cooperators and S agents bear an agent cost.

Red area: Selfish risk-seeking agents, **Yellow** area: Generous risk-averse agents.

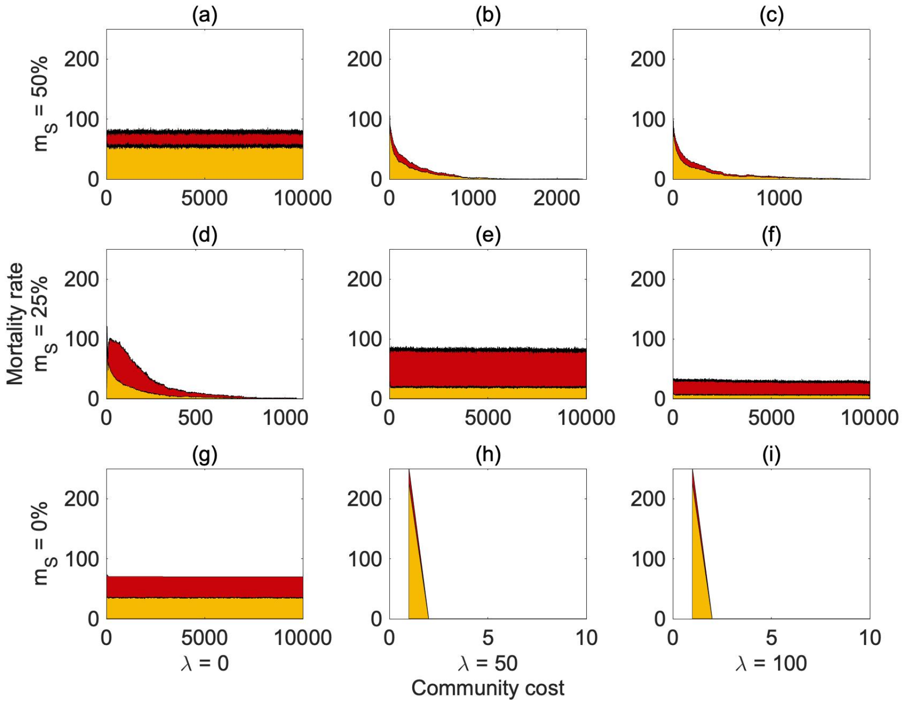

Figure S27: Evolution of the population over generations in an abundant environment ($e = 4.5$) when G agents act as conditional cooperators and S agents bear an individual cost.

Red area: Selfish risk-seeking agents, **Yellow** area: Generous risk-averse agents.

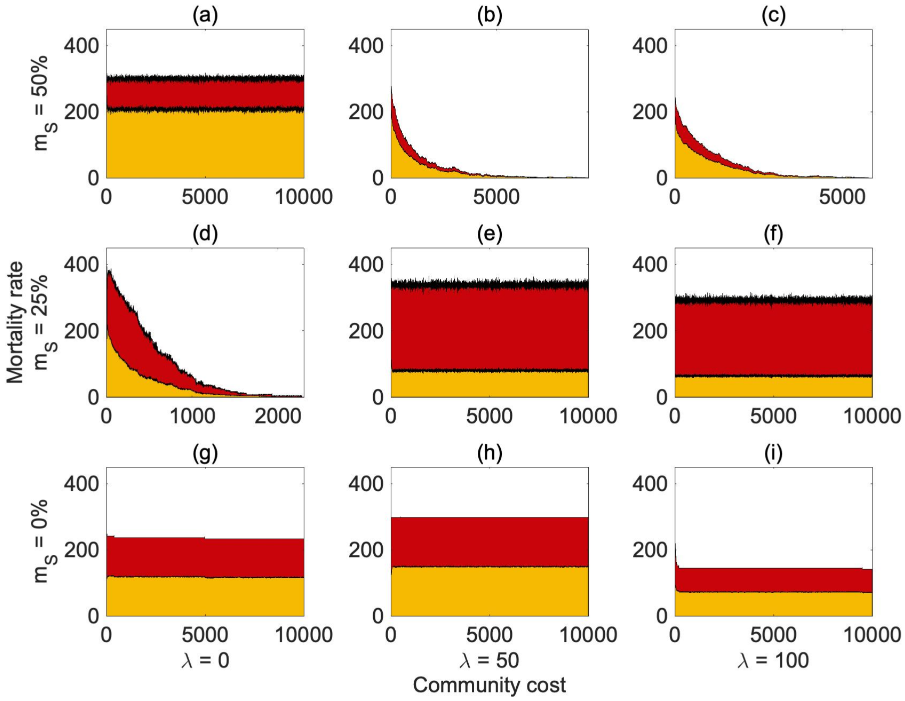

Figure S28: Evolution of the combined reproduction rates $\varphi_{S,S}, \varphi_{G,G}$ over generations in a scarce environment ($e = 1.2$) when G agents act as conditional cooperators and S agents bear an individual cost. **Yellow** line: $\varphi_{G,G}$, **Red** line: $\varphi_{S,S}$.
$\varphi_{G,G} = 1(0)/\varphi_{S,S} = 1(0)$ means that a generous risk-averse/selfish risk-seeking agent has a 100(0)% chance of reproducing a generous risk-averse/selfish risk-seeking offspring.

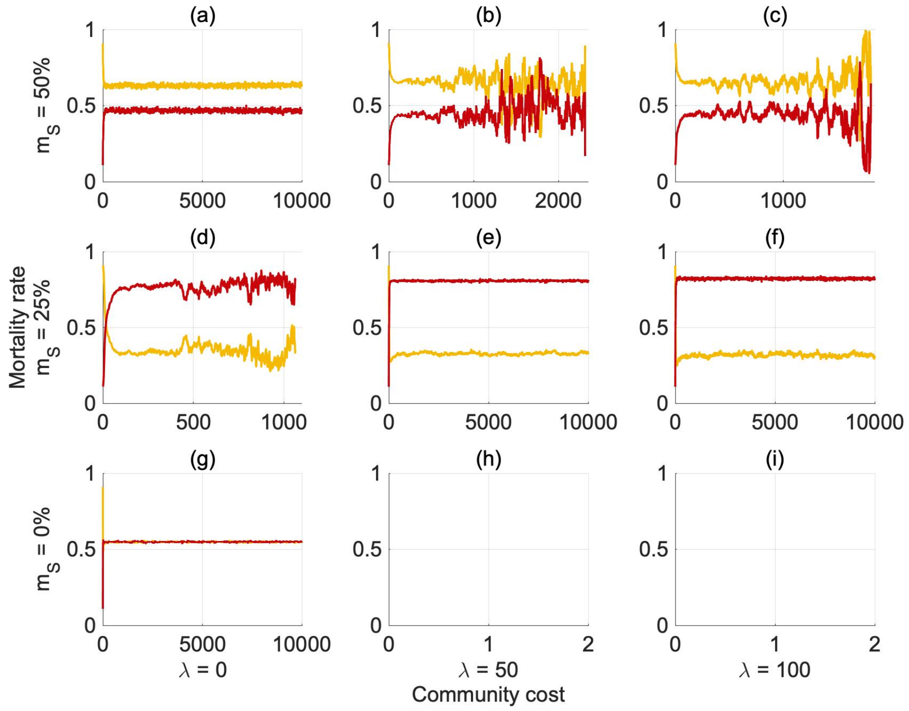

Figure S29: Evolution of the separate parameters (ω, γ and ϕ) regulating the reproduction mechanism (eq.7-11) over generations in a scarce environment ($e = 1.2$), when G agents act as conditional cooperators and S agents bear an individual cost.

Red line: γ, contribution of selfish risk-seeking parent to the probability of reproducing selfish risk-seeking offspring,

Yellow line: ω, contribution of generous risk-averse parent to the probability of reproducing generous risk-averse offspring,

Black line: ϕ, probability that agents will reproduce selfish risk-seeking offspring, independent of their own phenotype.

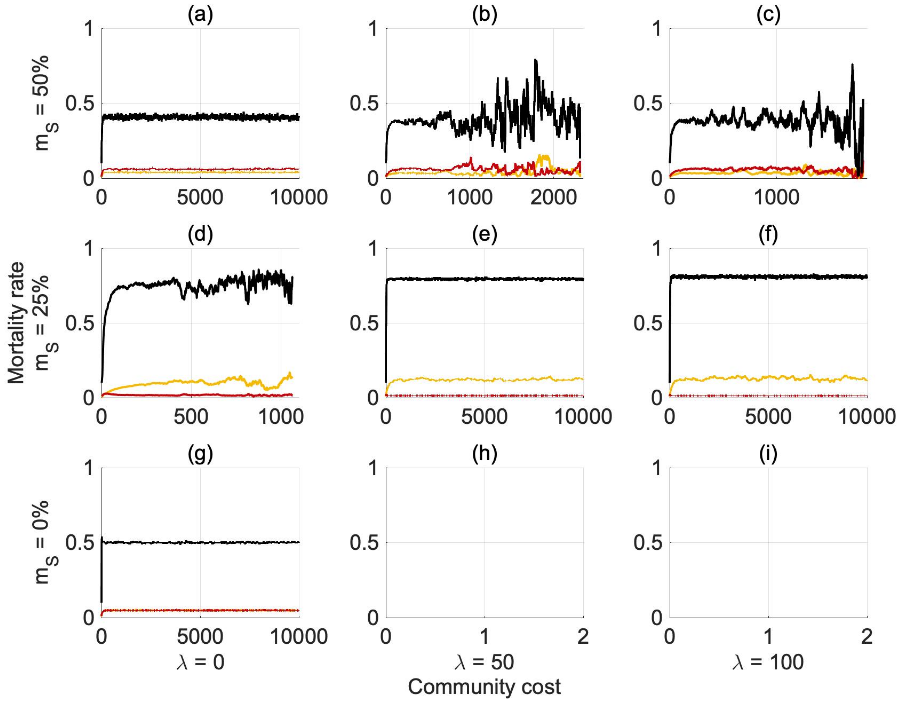

Figure S30: Evolution of the separate parameters (ω, γ and ϕ) regulating the reproduction mechanism (eq. 7-11) over generations in an abundant environment ($e = 4.5$), when generous risk-averse agents act as conditional cooperators and selfish risk-seekers bear an individual costs.

Red line: γ, contribution of selfish risk-seeking parent to the probability of reproducing selfish risk-seeking offspring,

Yellow line: ω, contribution of generous risk-averse parent to the probability of reproducing generous risk-averse offspring,

Black line: ϕ, probability that agents will reproduce selfish risk-seeking offspring, independent of their own phenotype.

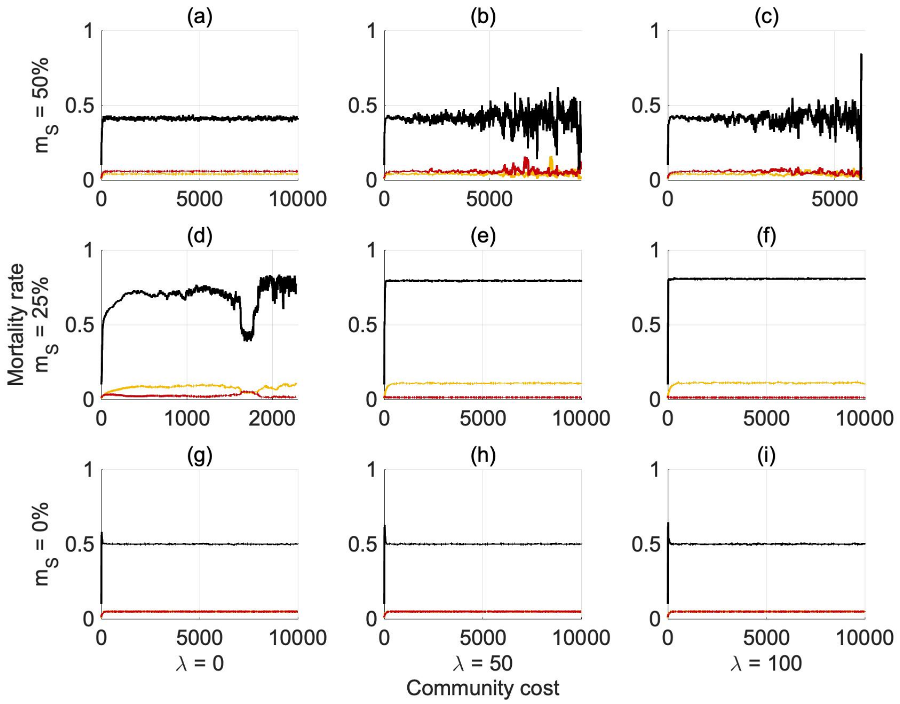

References

[1] L. W. Hyde, R. Waller, C. J. Trentacosta, D. S. Shaw, J. M. Neiderhiser, J. M. Ganiban, D. Reiss, L. D. Leve, Heritable and nonheritable pathways to early callous-unemotional behaviors, American Journal of Psychiatry 173 (9) (2016) 903–910.

[2] C. Tuvblad, S. Bezdjian, A. Raine, L. A. Baker, The heritability of psychopathic personality in 14-to 15-year-old twins: A multirater, multimeasure approach., Psychological assessment 26 (3) (2014) 704.
